# Long non-coding RNAs as molecular links and circulating biomarkers between type 2 diabetes and colorectal cancer: focus on shared signaling pathways, epigenetic regulation, and ubiquitination mechanisms

**DOI:** 10.3389/fmolb.2026.1775065

**Published:** 2026-05-21

**Authors:** Lang Wu, Qing Meng, Yang Zhou

**Affiliations:** 1 Dazhou Vocational and Technical College, Dazhou, Sichuan, China; 2 Sichuan Provincial People’s Hospital Chuan Dong Hospital, Dazhou First People’s Hospital, Dazhou, Sichuan, China; 3 Experimental Research Center, Foshan Hospital Affiliated to Southern Medical University, Foshan, China

**Keywords:** circulating biomarkers, colorectal cancer, epigenetic and ubiquitination regulation, long non-coding RNAs, molecular pathway, therapeutic targets, type 2 diabetes

## Abstract

Type 2 Diabetes (T2D) and Colorectal Cancer (CRC) share a complex bidirectional relationship driven by common metabolic and inflammatory pathways. This review comprehensively examines the pivotal role of Long Non-Coding RNAs (lncRNAs) as molecular bridges between T2D and CRC, regulating gene expression at chromatin, transcriptional, and post-transcriptional levels. We focus on specific lncRNAs including H19, ANRIL, KCNQ1OT1, UCA1, GAS5, MIR31HG, HNF1A-AS1, and MALAT1, which modulate shared oncogenic and metabolic signaling cascades such as PI3K/AKT, Wnt/β-catenin, NF-κB, and HIF-1α. Furthermore, we expand the scope beyond isolated lncRNA regulation to emphasize the lncRNA-miRNA crosstalk and the systemic involvement of the cardiovascular system. Recent evidence highlights that miR-217, miR-122, and the NBAT1/miR-21 axis are critical regulators not only in CRC progression but also in myocardial injury associated with T2D. Consequently, we propose that a holistic biomarker strategy must integrate panels of both lncRNAs and miRNAs to capture the full spectrum of metabolic, oncogenic, and cardiac risks. This updated perspective underscores the translational potential of targeting multi-ncRNA networks for early diagnosis, prognosis, and therapeutic intervention in patients with multimorbidity.

## Introduction

Type 2 diabetes (T2D) and colorectal cancer (CRC) are significant global health burdens that increasingly demonstrate deep molecular, metabolic, and clinical interconnections (Ottaiano et al., 2022; Bitaraf et al., 2025). Individuals with T2D exhibit a markedly elevated risk of developing CRC, driven by chronic hyperinsulinemia, systemic inflammation, oxidative stress, and metabolic dysregulation that collectively create a tumor-promoting microenvironment (Lawler et al., 2023; Zhang Y.Y. et al., 2024). At the same time, CRC itself can worsen systemic glucose homeostasis and insulin resistance (Samuel et al., 2025). Despite robust epidemiological associations, the molecular mechanisms linking these conditions remain insufficiently defined, limiting advances in early detection, risk prediction, and the development of tailored therapeutic strategies. LncRNAs have emerged as powerful integrators of metabolic and oncogenic signaling, exerting regulatory influence at transcriptional, post-transcriptional, and post-translational levels (Han et al., 2025). Their stability in circulation, tissue specificity, and sensitivity to metabolic stress highlight their potential as mechanistic drivers and clinically valuable biomarkers bridging T2D and CRC (Giordo et al., 2022). Recent evidence further suggests that this role extends beyond the immediate CRC-T2D axis to encompass systemic cardiovascular complications. For instance, studies by Zaafan et al. (Zaafan and Abdelhamid, 2025) and Abdel-Nasser et al. (Abdel-Nasser et al., 2023) have demonstrated that specific ncRNA pathways (e.g., miR-217, miR-122/Sirt-6/ACE2) are central to myocardial infarction pathogenesis, reinforcing the concept that lncRNAs act as systemic regulators linking metabolic, neoplastic, and cardiovascular pathologies.

Several key lncRNAs, including H19, ANRIL, KCNQ1OT1, UCA1, GAS5, MIR31HG, HNF1A-AS1, and especially MALAT1, converge on signaling networks shared by both diseases, such as PI3K/AKT/mTOR, NF-κB, Wnt/β-catenin, TGF-β/Smad, MAPK/ERK, STAT3, and HIF-1α (Zhao M. et al., 2021). Acting through competing endogenous RNA (ceRNA) mechanisms, chromatin remodeling, transcription factor sequestration, and regulation of ubiquitination-based protein turnover, these lncRNAs govern processes central to both metabolic dysfunction and tumorigenesis, including proliferation, apoptosis, angiogenesis, glycolysis, EMT, adipogenesis, β-cell function, and inflammatory signaling. MALAT1 exemplifies this dual involvement: in CRC, it enhances tumor growth, metastasis, and therapy resistance via β-catenin, AKT, and TGF-β pathways, as well as interactions with splicing regulators (Bitaraf et al., 2025; Xu et al., 2022). In T2D, MALAT1 drives endothelial dysfunction and microvascular injury through the activation of NF-κB, AGE-RAGE, and TGF-β, and influences insulin sensitivity and lipid metabolism (Abdulle et al., 2019; Ahmadi et al., 2024).

The clinical utility of these markers is further refined by the recognition of a genetic layer to their regulation. Recent findings by Ayeldeen et al. (2024) regarding the NBAT1/miR-21 axis highlight how PVT-1 polymorphisms can modulate miR-145 expression, suggesting that genetic variants significantly influence lncRNA-miRNA interactions and subsequent CRC progression, thereby necessitating a genotype-guided approach to biomarker interpretation.

Epigenetically, lncRNAs such as MALAT1, H19, ANRIL, and KCNQ1OT1 recruit chromatin-modifying complexes including PRC2, DNMT3A/B, and HDACs, altering gene expression in ways that promote carcinogenesis and diabetic complications. At the post-translational level, lncRNAs modulate ubiquitin-dependent protein stability by interacting with E3 ubiquitin ligases and deubiquitinases, affecting key metabolic and oncogenic proteins including HIF-1α, p53, NRF2, and HK2. From a clinical chemistry perspective, circulating and exosomal lncRNAs, including MALAT1, UCA1, KCNQ1OT1, H19, ANRIL, GAS5, and MIR31HG, represent promising non-invasive biomarkers for early CRC detection in diabetic patients, monitoring disease trajectory, predicting therapeutic responses, and evaluating metabolic deterioration, offering potential complementarity to routine laboratory markers such as HbA1c, C-peptide, CRP, IL-6, CEA, and CA19-9. The purpose of this review is to synthesize current evidence on circulating and tissue lncRNAs, including the central role of MALAT1, as shared molecular biomarkers linking T2D and CRC, with emphasis on epigenetic and ubiquitination-mediated mechanisms.

## Shared risk factors and pathways

CRC and T2D share many lifestyle and metabolic risk factors, as well as intersecting molecular pathways. Virtually all components of the metabolic syndrome predispose to both conditions. Obesity and sedentary lifestyle, for example, are strong risk factors for CRC and for diabetes (Yu et al., 2022; Grega et al., 2021). Adiposity promotes insulin resistance, hyperinsulinemia, and chronic inflammation, and obese individuals exhibit higher rates of CRC than lean persons. In the USA and other countries, obesity has been estimated to account for a significant fraction of CRC incidence (Bultman, 2017). Conversely, intentional weight loss and increased physical activity reduce both the incidence of diabetes and the risk of CRC (Zhang Y.Y. et al., 2024). Dietary patterns high in red and processed meat, animal fats, and refined sugars, characteristic of a “Western” diet, are linked to increased CRC risk (Bultman, 2017), and also promote insulin resistance and diabetes. In contrast, diets rich in fiber, fruits, vegetables, and whole grains are protective for CRC and help prevent T2D (Bultman, 2017). Tobacco smoking and excessive alcohol intake also raise CRC risk (by generating carcinogenic metabolites and promoting inflammation), and smoking is associated with higher diabetes risk; thus, behavioral factors further overlap. Importantly, even after adjusting for shared factors like obesity and inactivity, patients with diabetes have a higher CRC risk than non-diabetics, suggesting additional biological links (Yu et al., 2022).

At the cellular level, obesity and diabetes generate a pro-carcinogenic environment through chronic inflammation. Adipose tissue dysfunction in obesity leads to the elevation of pro-inflammatory cytokines (e.g., TNF-α, IL-6) and the activation of NF-κB signaling (Grega et al., 2021). These inflammatory mediators can act systemically and within the colonic mucosa to promote DNA damage, cell proliferation, angiogenesis, and tumorigenesis. In mouse models, a high-fat diet or genetic obesity increases CRC formation through inflammatory pathways (Garcia-Villatoro et al., 2020). In humans, a high body mass index is correlated with elevated COX-2 and prostaglandin levels (Zhang X. et al., 2011). Likewise, diabetes causes low-grade systemic inflammation that may potentiate tumor growth. Indeed, one review notes that “adipose tissue dysfunction has been associated with accentuated carcinogenesis with chronic subclinical inflammation” (Grega et al., 2021; Ruiz‐Malagón et al., 2025). Thus, inflammatory signaling is a shared mechanism linking metabolic disease to colorectal carcinogenesis.

The gut microbiome is another common link. Diet, obesity, and diabetes alter gut microbial composition (Liu S. et al., 2025). Some bacterial species enriched in individuals with diabetes or obesity are associated with an increased risk of CRC (Ruiz-Malagón et al., 2025; Chu, 2025). For example, the genus *Fusobacterium* is often increased in insulin-resistant individuals and can adhere to colonic epithelium, inducing pro-inflammatory and pro-survival signaling (Wu et al., 2022). Moreover, the microbiome in high-risk diets produces metabolites that modulate cancer risk (Mafe and Büsselberg, 2025; Modeel et al., 2026). Dietary fiber fermented by commensals yields short-chain fatty acids like butyrate that suppress tumors via epigenetic effects, including HDAC inhibition and anti-inflammatory actions (Bultman, 2017; Licciardi et al., 2011; Zhang Q. et al., 2025). Conversely, high-fat and red meat-enriched diets can stimulate the growth of bacteria that produce secondary bile acids and hydrogen sulphide, which can damage DNA (Zhang Q. et al., 2025; Diakité et al., 2022; Saha et al., 2024). Thus, diet/microbiome interactions influence both diabetes and CRC (Chu, 2025; Saha et al., 2024). Bultman (2017) highlighted that diet modulates the composition and function of gut microbiota, which influence inflammation and subsequently CRC, and that microbial metabolites are cofactors in epigenetic regulation. In individuals with diabetes, impaired microbiota diversity and increased endotoxemia may contribute to a tumor-promoting microenvironment (Chu, 2025). This evolving field suggests that microbiome changes represent a shared mechanistic pathway between diabetes and colorectal neoplasia (Grega et al., 2021; Mahboob et al., 2025).

At the level of signaling pathways and metabolism, insulin resistance, the hallmark of T2D, drives processes that also fuel cancer growth. Chronic hyperinsulinemia, the compensatory rise in circulating insulin accompanying insulin resistance, is strongly mitogenic (Caturano et al., 2025). Insulin, via the insulin receptor and hybrid insulin/insulin-like growth factor-1 (IGF-1) receptors, activates key oncogenic pathways (PI3K/AKT/mTOR, RAS/RAF/MEK/ERK) that promote cell proliferation and inhibit apoptosis (Caturano et al., 2025; Lecarpentier et al., 2017). Insulin also increases bioavailable IGF-1 by suppressing IGF-binding proteins (Sandhu et al., 2002). IGF-1 is a potent growth factor; *in vitro* and animal studies demonstrate that IGF-1 signaling enhances colorectal tumor cell growth and survival, often through the same PI3K/Akt and MAPK cascades (Sanchez-Lopez et al., 2016). Notably, colon cancers frequently overexpress the IGF-1 receptor and the fetal isoform of the insulin receptor, making them exquisitely responsive to circulating insulin/IGFs (Vigneri et al., 2015). Thus, hyperinsulinemia in diabetes may directly stimulate CRC development (Guraya, 2015). Indeed, insulin resistance and elevated C-peptide, a surrogate marker for insulin levels, have been associated with a higher risk of CRC in epidemiological studies (Guraya, 2015).

Importantly, the insulin/IGF axis and growth factor signaling also intersect with the canonical Wnt/β-catenin pathway, which is central to CRC pathogenesis (APC mutations in nearly all sporadic CRCs unleash β-catenin-driven proliferation) (Gligorijević et al., 2022). Recent work has shown that high glucose levels can activate Wnt signaling: elevated glucose induces β-catenin acetylation and nuclear translocation, thereby promoting Wnt target gene expression (Lecarpentier et al., 2017). In other words, the metabolic environment of diabetes may augment Wnt-mediated oncogenesis (Lecarpentier et al., 2017). Conversely, the nuclear co-activator PPARγ can inhibit β-catenin signaling, suggesting that metabolic therapies might modulate Wnt activity in the colon (Lecarpentier et al., 2017). Altogether, the insulin/IGF, PI3K/Akt, and Wnt/β-catenin pathways converge on the metabolic derangements of diabetes and the signaling abnormalities of CRC (Lecarpentier et al., 2017).

Hyperglycemia contributes additional pro-tumor effects (Li et al., 2019). High glucose generates reactive oxygen species (ROS) and advanced glycation end-products (AGEs), both of which damage DNA and promote inflammation (Chen Y. et al., 2024). In CRC patients, increased AGEs and activation of the receptor for AGEs (RAGE) have been linked to tumor invasion and metastasis via ERK/SP1/MMP2 cascades (Deng et al., 2017). These findings suggest chronic hyperglycemia in diabetes may accelerate CRC progression through non-insulin mechanisms as well (Zhao and Wu, 2024). Finally, diabetes and obesity cause epigenetic and metabolic reprogramming that can facilitate cancer (Kim and Scherer, 2021). For example, nutrient excess and metabolic stress in diabetes may alter DNA methylation and histone acetylation patterns in colonic cells (Ahmed et al., 2024), potentially silencing tumor-suppressor genes and activating oncogenes. The same dietary factors that feed tumors can also supply acetyl-CoA and other cofactors for epigenetic enzymes (Hao et al., 2022). A landmark review notes that dietary inputs and microbiota-derived metabolites, such as butyrate, act as cofactors for chromatin modifiers, influencing gene expression in ways that affect CRC risk (Wu et al., 2018). Likewise, the “Warburg effect” of cancer cells (aerobic glycolysis) is supported by hyperglycemia; abundant glucose in people with diabetes provides fuel for glycolytic metabolism, enabling rapid tumor growth (Burns and Manda, 2017; Joshi et al., 2015). Collectively, Tables 1, 2 summarize the central systemic mechanisms and intracellular signaling pathways that underlie the mechanistic overlap between diabetes and CRC.

**TABLE 1:** Shared signaling pathways and lncRNA regulation.

Pathway	Role in T2D	Role in CRC	Key lncRNAs involved
PI3K/AKT/mTOR	Insulin resistance, lipogenesis	Proliferation, survival	MALAT1, UCA1, KCNQ1OT1, GAS5
Wnt/β-catenin	β-cell function, adipogenesis	Stemness, EMT	MALAT1, MIR31HG, HNF1A-AS1, H19
NF-κB	Systemic inflammation	Tumor microenvironment	MALAT1, ANRIL, KCNQ1OT1
HIF-1α	Hypoxia in complications	Angiogenesis, glycolysis	H19, ANRIL, MIR31HG
TGF-β/Smad	Fibrosis in nephropathy	EMT, metastasis	KCNQ1OT1, MALAT1

**TABLE 2:** Epigenetic and Ubiquitination mechanisms of key lncRNAs linking type 2 diabetes and colorectal cancer.

LncRNA	Epigenetic mechanism	Ref.	Ubiquitination target/Effect	Ref.	Shared molecular outcome in T2D and CR
MALAT1	Recruits PRC2 (EZH2/SUZ12/EED) for H3K27me3 deposition; guides chromatin remodeling at target loci (e.g., E-cadherin, CDKN2A)	Abdel-Nasser et al. (2023), Arun et al. (2020), Amodio et al. (2018), Li et al. (2017), Zhang et al., 2020; Xu (2024), Gao et al. (2024)	Stabilizes SREBP-1c by inhibiting STUB1-mediated ubiquitination; protects FOXP3 from degradation	Vana et al. (2007), Wong et al. (2019)	Enhanced lipogenesis and inflammation (T2D); EMT and proliferation (CRC)
UCA1	Interacts with EZH2 for H3K27me3 on tumor suppressor genes (p21, p27); regulated by DNMT1-mediated promoter methylation	Craig-Schapiro et al. (2025), Li et al. (2014)	Facilitates Cbl-c-mediated degradation of GRK2, activating ERK/MMP9 signaling	Maghool et al. (2026)	Glycolytic reprogramming; sustained PI3K/AKT and inflammatory signaling
MIR31HG	Alters H3K4me3 and histone acetylation at promoters (e.g., FABP4); influences YY1-mediated transcriptional activation	Maeda et al. (2021)	Limited direct evidence; indirect via HIF-1α stabilization in hypoxia	Geng et al. (2024), Aghaei-Zarch (2024)	Warburg-like glycolysis; hypoxia adaptation and angiogenesis
KCNQ1OT1	Recruits PRC2 for H3K27me3; regulates imprinting via differential methylation at KvDMR locus (e.g., CDKN1C silencing)	Pan et al. (2021), Wang et al. (2017)	Stabilizes HK2 by preventing ubiquitin-dependent degradation; upregulates USP22 to stabilize PD-L1	Pan et al. (2021), Duan et al. (2020)	Aerobic glycolysis; immune evasion and fibrosis/EMT
GAS5	Prevents excessive EZH2-mediated H3K27me3 silencing of tumor suppressors; promotes E2F1 binding to CDKN1B promoter	Bai et al. (2023)	Binds YAP WW domain, promoting phosphorylation and subsequent ubiquitin-proteasome degradation	Xia et al. (2022)	Suppression of oncogenic proliferation; enhanced insulin signaling
HNF1A-AS1	Binds EZH2 and forms RNA–DNA triplexes for H3K27me3; scaffolds PRMT1 for arginine methylation	Yang et al. (2018a), Xian et al. (2021)	Blocks TRIM25-mediated ubiquitination of HNF1A protein, stabilizing it; promotes CDC34-mediated p21 degradation	Xian et al. (2021), Wang et al. (2019)	Metabolic gene activation (T2D); cell-cycle progression and Wnt activation (CRC)
H19	Recruits PRC2/EZH2 and MBD1 to imprinted loci/DMRs; influences histone modifications and DNA methylation (H19/IGF2 locus)	Beucher et al. (2022), Yang et al. (2014)	Inhibits VHL-mediated ubiquitination of HIF-1α under hypoxia; modulates stability via interactions with E3 ligases	Liu et al. (2018), Liu et al. (2020), DeForest et al. (2023)	Hypoxia-driven angiogenesis and glycolysis; insulin/let-7 dysregulation
ANRIL	Recruits PRC2 (EZH2) and PRC1 (CBX7) for H3K27me3 and H2AK119ub at CDKN2A/B locus; cooperates with DNMT3B for DNA methylation	Huang et al. (2018), Li et al. (2025), Li et al. (2016)	Indirectly disrupts p53/MDM2 balance; influences ubiquitin pathways via repressed ARF	Rajendran et al. (2024)	Cell-cycle dysregulation; repression of tumor suppressors and metabolic regulators

The epidemiological link between diabetes and CRC has significant clinical consequences (Yu et al., 2022). Numerous cohort and case-control studies consistently show that T2D is associated with a modestly higher CRC risk (Yu et al., 2022; Guan et al., 2025). Meta-analyses report that diabetes confers roughly a 30%–40% increased risk of colon cancer compared to non-diabetics (Guraya, 2015). The excess risk appears partly independent of shared factors, such as obesity or age, suggesting that diabetes itself (or its metabolic sequelae) contributes causally to CRC. Diabetic patients are also diagnosed at more advanced stages and have worse survival: CRC patients with diabetes have lower 5-year survival and higher mortality than non-diabetic CRC patients (Bella et al., 2013). Thus, diabetes not only predisposes to cancer development but may worsen outcomes.

**FIGURE 1:**
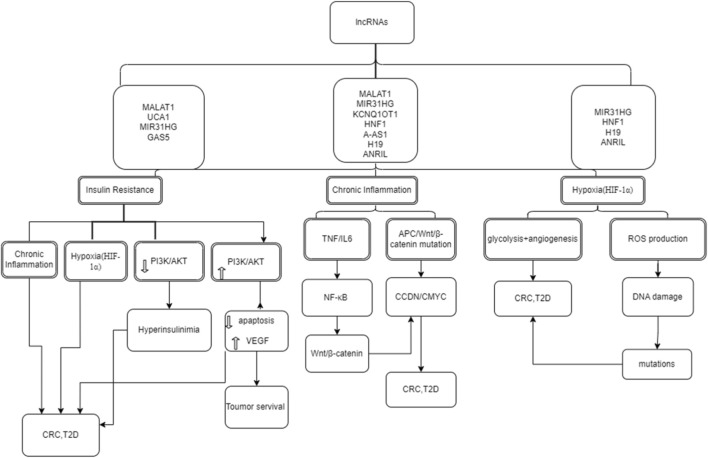
Schematic overview of the cross-talk between metabolic (T2D) and oncogenic (CRC) signaling pathways modulated by lncRNAs. This diagram illustrates the convergence of shared molecular hubs, including PI3K/AKT, Wnt/β-catenin, MAPK/ERK, NF-κB, TGF-β/Smad, and HIF-1α. The figure highlights how specific lncRNAs act as central regulators, integrating systemic metabolic stress (e.g., hyperglycemia, insulin resistance) with tumor-specific oncogenic drivers to promote dual pathology.

LncRNAs are transcripts longer than 200 nucleotides that, despite lacking protein-coding potential, are potent regulators of gene expression (Saw and Song, 2025). These molecules interact with chromatin modifiers, transcription factors, and microRNAs (miRNAs), influencing epigenetic states, RNA stability, and signaling pathways (Saw and Song, 2025). Dysregulated lncRNAs have been repeatedly linked to cancer and metabolic diseases, reflecting their broad impact on cellular physiology (Figure 1) (Adjeroh et al., 2024; Shabna et al., 2023). Indeed, DM is an independent risk factor for CRC, suggesting that it shares overlapping molecular drivers (Table 1) (Shabna et al., 2023). Many lncRNAs implicated in CRC also modulate glucose homeostasis or insulin signaling; for example, MALAT1 is overexpressed in diverse cancers (including CRC) and in diabetic tissues, where it alters insulin sensitivity via NRF2 and JNK pathways (Shabna et al., 2023).

This extensive crosstalk is now being mapped onto systemic comorbidities. Recent research emphasizes that ncRNA dysregulation extends to cardiovascular health, with specific miRNAs (like miR-217) acting as biomarkers for myocardial infarction (Zaafan and Abdelhamid, 2025), and axes like miR-122/Sirt-6/ACE2 confirming ncRNA involvement in cardiac injury (Abdel-Nasser et al., 2023). Furthermore, genetic variation is a key modulator of this landscape; polymorphisms in PVT-1 are shown to alter the expression of miR-145, which impacts the NBAT1/miR-21 axis in CRC (Ayeldeen et al., 2024), underscoring the potential for genotype-guided therapy.

Collectively, these findings suggest extensive crosstalk between CRC and diabetes via lncRNAs. Many shared lncRNAs regulate chromatin and signaling pathways relevant to both glucose metabolism and tumor growth (Wen et al., 2022). They can alter DNA methylation or histone marks and can interface with ubiquitination (Huang et al., 2022). This study focuses on 8 lncRNAs implicated in both diseases, aiming to characterize their role in epigenetic regulation and ubiquitination, and to map their convergent pathways (Table 2). Their frequent detection in accessible biofluids and tissues further highlights their translational potential as biomarkers and therapeutic targets. Investigating these common lncRNAs offers a unique opportunity to uncover unifying disease mechanisms and develop targeted strategies for both metabolic and neoplastic pathologies.

## MALAT1

LncRNAs have become increasingly relevant to clinical chemistry because of their remarkable stability, disease-specific expression, and ability to regulate diverse metabolic and oncogenic pathways. Among these molecules, Metastasis-Associated Lung Adenocarcinoma Transcript 1 (MALAT1/NEAT2) is one of the earliest-discovered and most extensively studied nuclear lncRNAs (Tiansheng et al., 2020; Arun et al., 2020). MALAT1 is a ∼8-kb transcript located on chromosome 11q13 and is evolutionarily conserved across mammals (Arun et al., 2020). It localizes primarily to nuclear speckles, where it modulates gene expression by scaffolding nuclear proteins, regulating transcription, mediating epigenetic remodeling, and influencing alternative splicing (Arun et al., 2020). Although MALAT1 is not required for normal development, its aberrant upregulation is consistently observed in CRC and T2D, two complex disorders characterized by chronic inflammation, metabolic stress, and dysregulated cellular signaling (Xu et al., 2022; Arun et al., 2020). Increasing evidence indicates that MALAT1 sits at the intersection of metabolic, inflammatory, and oncogenic pathways, positioning it as a potential diagnostic biomarker and therapeutic target in clinical laboratory medicine (Xu et al., 2022; Arun et al., 2020).

MALAT1 is markedly overexpressed in CRC tissues, circulating plasma, and metastatic sites, where elevated levels correlate with advanced TNM stage, lymph node metastasis, distant spread, reduced survival, and diminished response to oxaliplatin-based chemotherapy (Uthman et al., 2021; Jia et al., 2022). These consistent clinical associations highlight MALAT1’s utility as both a prognostic marker and a potential predictor of therapeutic response.

A central oncogenic mechanism of MALAT1 is its function as a ceRNA (Xu et al., 2022). MALAT1 sequesters several tumor-suppressive miRNAs, thereby enhancing the expression of their oncogenic targets. For example, MALAT1 sponge miR-363-3p to promote EZH2 upregulation, driving the repression of E-cadherin and facilitating the EMT (Amodio et al., 2018). It also binds to miR-218, promoting metastatic behavior (Li et al., 2017), and indirectly derepresses SOX9 by competing with miR-145, thereby enhancing CRC cell proliferation (Arun et al., 2020). Through these ceRNA interactions, MALAT1 influences key oncogenic signaling pathways, including the Wnt/β-catenin, Hippo/YAP, PI3K/AKT/mTOR, and VEGF signaling pathways, pathways that can be quantitatively assessed in clinical molecular diagnostics (Bitaraf et al., 2025; Zhang et al., 2020).

Epigenetic remodeling represents another central mechanism by which MALAT1 drives CRC progression. MALAT1 interacts with components of the polycomb repressive complex 2 (PRC2), including EZH2, SUZ12, and EED, guiding the complex to specific genomic loci and increasing histone H3K27 trimethylation (Xu et al., 2022; Amodio et al., 2018). This epigenetic repression targets genes such as CDKN2A (Xu, 2024), MYT1 (Chow and Poon, 2013; Vana et al., 2007), HOXC8 and HOXA9 (Osmond et al., 2022), and E-cadherin (Amodio et al., 2018), resulting in enhanced cell-cycle progression, EMT, and invasive capacity. Because PRC2-mediated chromatin signatures are readily measurable, MALAT1-associated epigenetic patterns may support the development of novel diagnostic and prognostic assays. In addition to transcriptional control, MALAT1 affects RNA-processing pathways. By modulating interactions within the SFPQ/PTBP2 complex, MALAT1 influences alternative splicing events that promote oncogenic gene expression programs (Xu et al., 2022). This capacity to rewire the transcriptome further reinforces its central regulatory role in CRC biology.

Single-cell RNA sequencing is uncovering just how differently MALAT1 behaves depending on the cell type and disease setting. For instance, in gastric cancer (GC), integrated analysis of 119,878 single cells across different TCGA molecular subtypes revealed that MALAT1 functions as a subtype-specific regulator, forming a regulatory pair with CTNNB1 (β-catenin) specifically in the chromosomal instability (CIN) subtype, while also serving as a prognostic marker for genomically stable tumors (Ren et al., 2025). Meanwhile, in colorectal cancer, scRNA-seq of 75,373 tumor-infiltrating lymphocytes from both untreated and immunotherapy-treated patients demonstrated that MALAT1 is highly expressed in specific immune subsets, particularly a distinct population of regulatory T cells characterized as MALAT1 high Tregs and M2 macrophages. Interestingly, when researchers used PD-1 checkpoint blockers in mouse models, those MALAT1-high regulatory T cells (Tregs) and M2 macrophages dropped dramatically, linking MALAT1-expressing immune cells to immunotherapy response (Kim et al., 2021). Beyond immune cells, MALAT1 is overexpressed 2.26-fold in CRC tumor cells compared to non-cancerous tissues and correlates with advanced TNM stage, lymph node metastasis, and reduced survival (Bitaraf et al., 2025). The Gao et al. study demonstrated that CD95 promotes CRC stemness through MALAT1 upregulation, with MALAT1 knockdown inhibiting CD95-induced tumorsphere formation and chemotherapy resistance, confirming a cell-autonomous role in cancer stem cell biology. Highlighting the power of scRNA-seq to uncover lncRNA heterogeneity that correlates with clinical outcomes (Gao et al., 2024).

In the pancreas, single-cell atlases initially suggested that MALAT1 is one of the most broadly expressed genes across different cell types (Wong et al., 2019; Luo et al., 2023). But after carefully filtering out background RNA signals, it became clear that this broad detection was partly due to technical noise, underscoring how MALAT1 is so ubiquitous that it’s often mistaken for a housekeeping gene (Craig-Schapiro et al., 2025). Still, its expression levels do seem to matter for islet health. Higher levels of certain MALAT1 variants were linked to better-functioning, more viable human islets, with implications for β-cell insulin content. This hints that healthy beta cells likely express more MALAT1 (Wong et al., 2019).

In T2D, scRNA-seq analysis of 4,990 beta cells revealed that MALAT1 is upregulated in beta cells with high ferroptosis and dedifferentiation scores (Ma et al., 2024). The study identified two β-cell clusters (Cluster 2 and Cluster 4) with pronounced ferroptosis features, and pseudo-time trajectory analysis showed that ferroptosis-related changes occur along the dedifferentiation trajectory. MALAT1 was identified in a ceRNA network potentially regulating ferroptosis-related genes.

In the intestinal epithelium, MALAT1 is one of the most highly expressed lncRNAs in both small intestine and colon epithelium, with functional studies confirming its role in intestinal epithelial cells where it regulates anti-microbial responses and maintains barrier integrity (Long et al., 2023). GRID-seq revealed MALAT1 binding to chromatin regulatory elements in epithelial cells, including promoters, intragenic regions, and distal elements. ATAC-seq showed that MALAT1-occupied regions lie within both open and closed chromatin in a gene-specific manner indicating of the epigenetic regulatory role of this lncRNA. Furthermore, if we go beyond single-cell approaches, MALAT1 expression is significantly elevated in the blood and affected tissues of individuals with diabetes, including endothelial cells, the retina, the kidney, the myocardium, and the liver, the major organs involved in diabetic complications (Abdulle et al., 2019; Huang et al., 2020; Yan et al., 2016). Upregulation is associated with increased inflammatory markers and endothelial dysfunction and is a risk factor for microvascular complications.

Hyperglycemia induces MALAT1 expression, which in turn promotes inflammatory cytokine production via NF-κB activation through the MyD88/IRAK1/TRAF6 axis (Abdulle et al., 2019; Huang et al., 2020). MALAT1 also modulates the SOCS3/JAK/STAT pathway by sponging miR-361-3p, enhancing IL-6 and TNF-α production (Arun et al., 2020). Knockdown studies consistently show reduced cytokine release, underscoring MALAT1’s role in diabetic vascular inflammation.

In metabolic tissues, MALAT1 contributes to insulin resistance by stabilizing SREBP-1c, preventing its ubiquitin-dependent degradation, and enhancing lipogenesis, hepatic lipid accumulation, and gluconeogenic gene expression (Yan et al., 2016). Beyond metabolic dysfunction, MALAT1 plays significant roles in well-characterized diabetic complications, including retinopathy, nephropathy, and cardiomyopathy (Perisset et al., 2023; Yang et al., 2022; Zheng et al., 2025).

In colorectal cancer and GC, exosomal MALAT1 from tumor-associated macrophages interacts with δ-catenin, preventing its ubiquitination and degradation by β-TRCP, leading to pathway activation and enhanced glycolysis-dependent proliferation (Bitaraf et al., 2025). Furthermore, The SENP8 identified in intestinal epithelial cells as a MALAT1 target is a SUMO peptidase involved in neddylation/deneddylation pathways, which are closely related to ubiquitination (Long et al., 2023). SENP8 levels predicted colon adenoma patient overall survival and disease-free survival, directly linking a MALAT1 target involved in protein modification pathways to clinical outcomes in CRC.

Several MALAT1-regulated mechanisms overlap across CRC and diabetes, highlighting its role as a central regulator of inflammation, metabolic stress, and cell fate decisions. Inflammation forms the most substantial mechanistic overlap. In CRC, MALAT1 interacts with NF-κB-p65 (Gupta et al., 2020), whereas in diabetes, it activates NF-κB through upstream innate immune signaling (Abdulle et al., 2019; Huang et al., 2020). Angiogenesis represents another convergence point: MALAT1 promotes VEGF signaling in CRC through YAP (Arun et al., 2020) and in diabetic retinopathy (DR) by stabilizing HIF-1α (Perisset et al., 2023). PI3K/AKT/mTOR signaling is active in both diseases, supporting tumor growth in CRC and insulin resistance in diabetes (Xu et al., 2022; Arun et al., 2020). MALAT1 also participates in epigenetic repression via PRC2 (Amodio et al., 2018) and regulates ubiquitination by stabilizing proteins such as FOXP3 (Li et al., 2021) and SREBP-1c (Yan et al., 2016).

MALAT1’s interactions with PRC2 contribute to widespread chromatin remodeling, leading to repression of tumor suppressors, dysregulated differentiation, and enhanced inflammatory gene expression (Garcia-Villatoro et al., 2020; Liu S. et al., 2025; Licciardi et al., 2011; Zhang Q. et al., 2025; Diakité et al., 2022; Saha et al., 2024). Ubiquitin-dependent protein stabilization adds a further layer of complexity: MALAT1 prevents STUB1-mediated degradation of FOXP3 in cancer (Li et al., 2021) and protects SREBP-1c in metabolic tissues (Yan et al., 2016). These epigenetic and post-translational mechanisms are increasingly measurable using modern molecular laboratory technologies, supporting the translational relevance of MALAT1.

## UCA1

Urothelial cancer-associated 1 (UCA1) is an lncRNA first identified in bladder cancer (Neve et al., 2018). UCA1 has three transcript isoforms (≈1.4, 2.2, and 2.7 kb), of which the 1.4-kb form is the most studied. UCA1 is highly expressed in many malignancies, including CRC, and generally correlates with aggressive phenotypes (Neve et al., 2018; Liu Z. et al., 2021). Mechanistically, UCA1 can act as an oncogene by influencing transcription and chromatin structure, interacting with chromatin modifiers (e.g., PRC2/EZH2), and functioning as a ceRNA that sponges miRNAs (Neve et al., 2018). UCA1 is also emerging as a key regulator of cell growth and metabolism (Liu Z. et al., 2021). These diverse functions make UCA1 a central player in diseases ranging from cancer to metabolic disorders (Wang et al., 2015; Maghool et al., 2026; Luan et al., 2020; Li et al., 2014).

UCA1 is an lncRNA frequently upregulated in CRC, where it functions as an oncogene (Liu S-J. et al., 2021). Clinically, high UCA1 expression in CRC tissues is associated with chemoresistance, metastasis, and a poor prognosis (Neve et al., 2018). Importantly, single-cell transcriptomic analyses reveal that UCA1 expression in CRC is not confined to malignant epithelial cells but is also detectable in stromal and immune cell populations within the tumor microenvironment, including T cells and plasma cells (Maghool et al., 2026). This broad cellular distribution suggests UCA1 may have paracrine or immunomodulatory roles alongside its cell-intrinsic oncogenic functions. Mechanistically, UCA1 acts primarily via ceRNA networks and epigenetic regulation (Ramli et al., 2021; Luan et al., 2020). UCA1sponges miR-143, derepressing oncogenic targets such as MYO6 and KRAS to drive proliferation and invasion (Li et al., 2014). Similarly, UCA1 binds to miR-28-5p, increasing the levels of BCL2, RAB22A, HOXB3, and CREB1, thereby promoting cell survival and chemoresistance (Liu Z. et al., 2021; Cui et al., 2019). The immune cell expression of UCA1 is particularly relevant as it correlates with an immunosuppressive microenvironment, including associations with Tregs and immune checkpoint molecules, potentially contributing to immune evasion in CRC (Maghool et al., 2026). UCA1 also modulates multiple signaling pathways, including the Wnt/β-catenin, MAPK, PI3K/AKT, JAK/STAT, and NF-κB pathways, contributing to tumor growth, survival, and drug resistance (Liu Z. et al., 2021). Clinically, plasma and tissue UCA1 are being explored as potential biomarkers for CRC, with elevated levels correlating with disease severity (Maeda et al., 2021).

In diabetes and metabolic disorders, UCA1 shows context-dependent roles (Geng et al., 2024). Paradoxically, UCA1 is often downregulated in T2DM, and low levels are associated with insulin resistance and higher cardiovascular risk (Geng et al., 2024; Huang S. et al., 2023). In skeletal muscle, UCA1 forms a ceRNA network with miR-143-3p and FGF21 (Huang S. et al., 2023). Under lipotoxic stress, decreased UCA1 and FGF21 impair mitochondrial function, whereas UCA1 overexpression restores FGF21 levels, reduces ROS, increases ATP production, and maintains metabolic homeostasis (Huang S. et al., 2023). In diabetic nephropathy (DN), UCA1 may inhibit the PI3K/AKT signaling pathway to protect kidney function (Aghaei-Zarch, 2024). In contrast, in DR, UCA1 is upregulated by high glucose in retinal endothelial cells, thereby sponging miR-624-3p and increasing VEGF-C, which promotes pathological angiogenesis (Yan et al., 2021). Thus, UCA1 exerts tissue-specific effects in diabetes: protective in muscle and kidney but pathogenic in retinal vessels.

Several molecular pathways regulated by UCA1 are shared between CRC and diabetes. Central to both is glucose metabolism: in cancer, UCA1 promotes glycolysis via the mTOR/STAT3 and HK2 pathways, while in muscle, it supports energy production via FGF21, both processes involving the UCA1/miR-143 axis (Ramli et al., 2021; Yan et al., 2021). UCA1 also influences PI3K/AKT, JAK/STAT, NF-κB, Wnt/β-catenin, and angiogenic VEGF signaling, which are critical in tumor growth, insulin resistance, inflammation, and diabetic microvascular complications (Liu Z. et al., 2021). Mechanistically, UCA1 may participate in a biphasic response: early diabetes-associated UCA1 suppression impairs metabolism, while chronic oxidative stress in advanced disease may induce UCA1 overexpression, potentially creating a tumor-permissive environment. The single-cell expression profile of UCA1 in CRC, spanning epithelial, stromal, and immune compartments, suggests its dysregulation could similarly affect multiple cell types in the diabetic milieu. For instance, its expression in immune cells hints at a potential direct role in modulating the chronic low-grade inflammation characteristic of T2DM, which in turn fuels cancer progression. Future spatial transcriptomic studies could map UCA1 expression relative to specific niches like islets, adipose tissue, or diabetic kidney glomeruli, to clarify its cell-type-specific functions in metabolic disease and its connection to the pro-tumorigenic microenvironment.

Epigenetically, UCA1 is regulated by DNMT1-mediated promoter methylation and chromatin remodeling (Pan et al., 2021). In turn, UCA1 recruits EZH2 to deposit H3K27me3 on tumor suppressor genes, including p21 (CDKN1B), p27 (CDKN1B), and miR-143, suppressing cell-cycle inhibitors and promoting proliferation (Liu Z. et al., 2021). UCA1 also interacts with chromatin remodelers such as BRG1 and CTCF to modulate β-catenin transcription (Neve et al., 2018; Neve et al., 2025). These epigenetic effects may also influence metabolic genes in diabetes, though direct evidence is limited. Post-translationally, UCA1 affects protein ubiquitination and stability; for instance, UCA1 facilitates Cbl-c-mediated GRK2 degradation (Wang et al., 2017), activating ERK/MMP9 signaling in cancer, with potential implications for insulin signaling in diabetes (Liu Z. et al., 2021; Shu et al., 2024). Collectively, UCA1 integrates transcriptional, epigenetic, and post-translational regulation to control pathways shared by CRC and diabetes, including metabolism, growth, angiogenesis, and inflammation.

## MIR31HG

MIR31HG (also called LncHIFCAR or LOC554202) is an lncRNA of ∼2.1 kb on chromosome 9p21.3 that hosts miRNA-31 in its second intron. Its aberrant expression in multiple cancers and diseases, such as psoriasis and IgA nephropathy, underscores its importance, inspiring researchers and clinicians to appreciate its potential in understanding disease mechanisms and developing treatments (Ruan et al., 2023).

MIR31HG has emerged as a critical regulator in CRC pathogenesis, exerting oncogenic effects by modulating multiple signaling pathways (Wang et al., 2022). Mechanistic studies suggest that MIR31HG primarily promotes tumor progression by interacting with the Wnt/β-catenin pathway, facilitating the nuclear translocation of β-catenin and activating downstream proliferative genes. In addition, MIR31HG enhances CRC aggressiveness by activating the PI3K–AKT–mTOR axis, thereby promoting cell survival, invasion, and resistance to therapeutic agents (Wang et al., 2022). A key aspect of its function is acting as a ceRNA, where it sequesters miR-361-3p, leading to the derepression of the transcription factor YY1 (Guo et al., 2021). This establishes a positive feedback loop, as YY1 further upregulates MIR31HG expression, amplifying its oncogenic effects. Through this axis, MIR31HG not only drives tumor proliferation but also enhances glycolytic metabolism and angiogenesis, which are critical hallmarks of CRC progression. Recent single-cell multiomic studies in non-cancerous disease contexts reveal important cell-type-specific insights that parallel its oncogenic roles. In Autosomal Dominant Polycystic Kidney Disease (ADPKD), single-nucleus RNA sequencing identified MIR31HG as a key marker specifically upregulated in pathogenic, cyst-lining collecting duct epithelial cell subpopulations (PKD-CDC1 and PKD-CDC2). Crucially, this upregulation was driven by a distal enhancer that became differentially accessible in the disease state, as revealed by integrated single-nucleus ATAC-seq epigenomic profiling (Muto et al., 2022). This finding demonstrates that MIR31HG dysregulation is not a uniform phenomenon but is orchestrated by precise epigenetic remodeling in specific pathogenic cell states.

Although MIR31HG is best studied in the context of cancer, it also modulates metabolic and diabetic processes (Guo et al., 2021). MIR31HG promotes adipogenesis: in human adipose-derived stem cells, overexpression of MIR31HG enhances adipocyte differentiation, whereas knockdown of MIR31HG blocks differentiation (Huang et al., 2017). This occurs via epigenetic regulation: loss of MIR31HG reduces H3K4me3 and H3 acetylation at the FABP4 promoter, suppressing its expression (Huang et al., 2017). MIR31HG also influences diabetic wound healing: exosomal MIR31HG regulates HIF-1α and boosts fibroblast proliferation in diabetic skin models (Shen et al., 2022). Additionally, MIR31HG is reduced in patients with diabetic neuropathy, suggesting its value as a circulating biomarker (Groeneweg et al., 2020). The ADPKD study further strengthens the link between MIR31HG and hypoxia-response pathways in a specific cellular niche. The MIR31HG-high cystic cells exhibited significant enrichment of glycolysis and hypoxic response gene signatures (Muto et al., 2022), directly tying MIR31HG expression to a metabolic shift akin to the Warburg effect. This provides a clear, single-cell-resolution example of MIR31HG’s role in driving a hypoxic and glycolytic cellular program outside of cancer.

The oncogenic functions of MIR31HG in CRC show notable convergence with molecular mechanisms relevant to diabetes, indicating that MIR31HG may influence disease processes shared across metabolic and oncogenic pathophysiology. In CRC, MIR31HG enhances glycolytic metabolism by upregulating glycolytic enzymes and promoting the Warburg effect, an effect parallel to the dysregulated glucose metabolism characteristic of diabetic tissues (Guo et al., 2021). MIR31HG also engages hypoxia-related signaling through its interaction with HIF-1α, a transcription factor activated in both the tumor microenvironment and diabetic wounds, where hypoxia plays a central role in impaired tissue repair. Angiogenic pathways represent another shared molecular axis: MIR31HG increases VEGFA expression and promotes tumor vascularization (Guo et al., 2021). At the same time, diabetic complications such as retinopathy and wound healing abnormalities are likewise marked by dysfunctional or excessive neovascularization. Moreover, MIR31HG contributes to chronic inflammation through SASP-associated IL1A regulation, mediated by YBX1, which mirrors the persistent low-grade inflammation observed in diabetes (Montes et al., 2021). These overlapping processes, glycolysis, hypoxia, angiogenesis, and inflammation, highlight the potential of MIR31HG as a molecular link between CRC and diabetes. However, despite compelling mechanistic parallels, direct experimental evidence confirming MIR31HG as a unified pathogenic driver across both diseases remains limited, underscoring the need for further integrative studies.

## KCNQ1OT1

KCNQ1OT1 (KCNQ1 opposite strand/antisense transcript 1) is a lncRNA located on chromosome 11p15.5 that plays a critical regulatory role in various cellular processes, including proliferation, EMT, apoptosis, autophagy, and inflammation via epigenetic regulation, ubiquitination-mediated protein stabilization, and miRNA sponging (Cagle et al., 2021). Its dysregulation has been implicated in multiple diseases, such as CRC, prostate cancer, diabetes, DN, and diabetic cardiomyopathy (DCM). Emerging evidence highlights KCNQ1OT1 as a key player in both CRC progression and diabetes-related complications, often through shared molecular pathways, including PI3K/AKT/mTOR, Wnt/β-catenin, NF-κB, Hippo, RAS/ERK, and TGF-β/Smad3 signaling. Elevated KCNQ1OT1 expression is associated with poor prognosis in CRC patients, underscoring its potential as a therapeutic target (Cagle et al., 2021; Duan et al., 2020; Lin et al., 2021).

Moving beyond bulk tissue analyses, snRNA-seq in neuroinflammatory disease models has revealed that KCNQ1OT1 expression is highly cell-type-specific. In a study of anti-NMDA receptor encephalitis, KCNQ1OT1 was identified as a key differentially expressed lncRNA specifically within hippocampal inhibitory neurons. Intriguingly, it exhibited opposite expression patterns in different disease models, upregulated in an active immunization model and downregulated in a passive model, highlighting its context-dependent regulation within a defined neuronal subset (Bai et al., 2023). This underscores the importance of dissecting its function at single-cell resolution, as its role can be masked or misinterpreted in bulk tissue samples.

In CRC, KCNQ1OT1 exerts its oncogenic effects through multiple mechanisms. It functions as ceRNA, sponging miRNAs to modulate downstream targets (Liu et al., 2024; Zhan et al., 2023). For instance, KCNQ1OT1 binds miR-34a, upregulating Atg4B and enhancing chemoresistance in colon cancer. Similarly, KCNQ1OT1 boosts MTX resistance in CRC cells by influencing PPP1R1B expression via miR-760 and through the cAMP pathway (Cagle et al., 2021; Xian and Zhao, 2019). Similarly, it promotes metastasis via the KCNQ1OT1/miR-484/ANKRD36 axis and drives tumor progression through the miR-181a-5p/PCGF2 pathway (Xia et al., 2022). It also leads to overexpression of ZNF146, a zinc finger protein increased in 80% of CRC patients, via sponging mir-216b-5p, further facilitating CRC progression (Zhu et al., 2021; Ferbus et al., 2003; Perez-Oquendo and Gibbons, 2022). Additionally, KCNQ1OT1 influences immune evasion by upregulating CD155, a ligand for TIGIT, which triggers CD8^+^ T-cell exhaustion. Knockdown of KCNQ1OT1 reduces CD155 levels, enhances CD8^+^ T-cell infiltration and IFN-γ production, and improves antitumor immunity. Furthermore, KCNQ1OT1 suppresses T-cell metabolism by impairing glucose uptake via the PIP3/AKT pathway, further contributing to immune suppression in the tumor microenvironment (Lin et al., 2021; Liu J. et al., 2021).

The tumor microenvironment (TME) of CRC is highly heterogeneous. While specific scRNA-seq data on KCNQ1OT1 in CRC cell types is limited, insights from other cancers suggest its expression is likely compartmentalized. For example, in GC, scRNA-seq analysis revealed that KCNQ1OT1 does not act as a broad marker but forms a subtype-specific regulatory network. It was identified as part of the KCNQ1OT1-CCND2 regulatory pair specifically within tumors of the Microsatellite Instability (MSI) molecular subtype (Ren et al., 2025). This suggests that in gastrointestinal cancers, KCNQ1OT1’s oncogenic role may be dominant within specific molecular or cellular subpopulations, such as MSI-high tumor epithelial cells, rather than being uniformly expressed across all cells. Investigating KCNQ1OT1 expression in CRC scRNA-seq datasets across epithelial, immune, and stromal compartments is a crucial future direction to spatially map its pro-tumorigenic functions.

In diabetes-related complications, KCNQ1OT1 exacerbates pathogenesis through similar ceRNA mechanisms. In DCM, it sponges miR-214-3p, increasing caspase-1 and TGF-β1/smad expression, which promotes cardiomyocyte apoptosis and fibrosis (Yang et al., 2018a). In DN, KCNQ1OT1 binds to miR-506-3p and miR-18b-5p, thereby upregulating SORBS2 and activating NF-κB, which leads to oxidative stress and renal damage. It also exacerbates renal fibrosis by sequestering miR-93-5p, leading to ROCK2 overexpression and excessive extracellular matrix (ECM) deposition (Zhao et al., 2021b). In DR, KCNQ1OT1 worsens disease progression by sponging miR-1470 and upregulating EGFR, while in cataracts, it promotes EMT via the miR-26a-5p/ITGAV/TGF-β/Smad3 axis (Xia et al., 2022).

The cell-specific dysregulation of KCNQ1OT1 in diabetic complications remains an open question. It is plausible that in DN, KCNQ1OT1 upregulation may be specific to injured podocytes or activated fibroblasts, while in retinopathy, it might be localized to Müller glia or retinal ganglion cells. Spatial transcriptomics could powerfully test these hypotheses by mapping KCNQ1OT1 expression to pathological lesions like glomerulosclerosis or retinal microaneurysms, directly linking its expression to zones of inflammation and fibrosis. This will dramatically increase the precision of targeting this lncRNA for therapeutic benefit.

The overlapping pathways regulated by KCNQ1OT1 in both CRC and diabetes highlight its dual role in disease progression. For example, PI3K/AKT/mTOR activation drives proliferation in CRC and insulin resistance in diabetes (Duan et al., 2020; Ramasubbu and Devi Rajeswari, 2023), while TGF-β signaling promotes fibrosis in DN and EMT in CRC metastasis. KCNQ1OT1 also upregulates NF-κB, mediating inflammation in diabetes and immune evasion in CRC (Xia et al., 2022; Zhao et al., 2021b). Additionally, it enhances Wnt/β-catenin signaling by stabilizing CTNNB1, further fueling cancer progression. Silencing KCNQ1OT1 has been shown to reduce oncogenic proteins (e.g., Bcl-2, MMP9, Cyclin D1) while increasing pro-apoptotic markers (e.g., cleaved Caspase-3, E-cadherin), suggesting its potential as a therapeutic target (Cagle et al., 2021; Lin et al., 2021).

KCNQ1OT1 also plays a critical role in post-translational regulation. In CRC, it stabilizes hexokinase 2 (HK2), enhancing aerobic glycolysis and tumor proliferation by preventing HK2 ubiquitination and degradation (Cagle et al., 2021). Additionally, KCNQ1OT1 sponges miR-30a-5p, leading to USP22 upregulation, a deubiquitinase that stabilizes PD-L1. This mechanism promotes immune evasion by suppressing the activity of CD8^+^ T cells (Xian et al., 2021). KCNQ1OT1 also modulates autophagy by increasing LC3 expression, contributing to chemoresistance (Lin et al., 2021). Epigenetically, KCNQ1OT1 regulates imprinted genes in the 11p15.5 region, such as CDKN1C, known as p57, a cell growth and division control protein, through differential methylation at the KvDMR locus. Additionally, it has been shown to have a correlation with CTNNB1, also known as beta-catenin, which leads to cancer progression through the activation of the beta-catenin pathway (Cagle et al., 2021; Lin et al., 2021). Research done on HCT116-siKCN and SW480-siKCN cells showed that silencing KCNQ1OT1 will result in slowed cell growth and fewer cells in G2/M phase via enriching Gene Ontology (GO) and Kyoto Encyclopedia of Genes and Genomes (KEGG) pathways, suggesting that KCNQ1OT1 can reduce the cell size and tumor progression (Liu et al., 2024). Polymorphisms (e.g., rs35622507) and promoter methylation further influence its expression, impacting chemoresistance in CRC and metabolic dysregulation in diabetes (Zhang and Wang, 2022).

In summary, KCNQ1OT1 is a multifunctional lncRNA that drives disease progression in CRC and diabetes through epigenetic regulation, miRNA sponging, and post-translational modifications. Its involvement in shared pathways underscores its dual role in cancer and metabolic disorders, making it a promising candidate for targeted therapies. Further research is needed to fully elucidate its mechanisms and explore its clinical applications.

## GAS5

Growth arrest-specific transcript 5 (GAS5) is a well‐studied lncRNA located at 1q25, a genomic region often disrupted in multiple cancer types and involved in cell growth, apoptosis, and stress responses (Nguyen et al., 2025; Shi et al., 2019). GAS5 is transcribed as a non-coding RNA containing snoRNAs, but owing to a stop codon, none of its transcripts are translated (Shi et al., 2019). GAS5 can act as a molecular decoy, scaffold, and miRNA sponge (Nguyen et al., 2025; Shi et al., 2019). Notably, GAS5 is generally considered a tumor suppressor: it is downregulated in many cancers, including breast, lung, gastric, and CRC, and its overexpression inhibits proliferation and metastasis (Nguyen et al., 2025; Xie et al., 2022). Emerging evidence also implicates GAS5 in metabolic regulation: reduced GAS5 expression is observed in T2DM patients, and GAS5 overexpression improves insulin secretion and sensitivity (Shi et al., 2019; Luo et al., 2020). Importantly, while bulk studies define these roles, emerging single-cell and spatial transcriptomic approaches are revealing that GAS5’s function is highly cell-type-specific and context-dependent within diseased tissues. Below, we review studies of GAS5 in CRC and diabetes, highlighting shared pathways (e.g., PI3K/AKT, Hippo/YAP) and its roles in epigenetic and ubiquitin‐mediated regulation.

In CRC, multiple studies find GAS5 downregulated and tumor‐suppressive. For example, Xie et al. (2022) observed significantly lower GAS5 expression in CRC tissues and cell lines, and showed GAS5 knockdown promoted CRC cell proliferation, migration, and xenograft tumor growth. Mechanistically, GAS5 sponges oncogenic miR-21, relieving miR-21’s repression of targets: GAS5 increases the expression of leukemia inhibitory factor receptor (LIFR) by binding miR-21, thereby promoting apoptosis. This GAS5 miR-21–LIFR axis was shown to inhibit CRC metastasis and proliferation. More generally, GAS5/miR-21 interactions regulate PI3K/AKT signaling in many cancers: GAS5 overexpression leads to PTEN upregulation (via miR-21 inhibition), reducing AKT/mTOR activity (Nguyen et al., 2025; Xie et al., 2022). Thus, GAS5 loss can activate AKT-driven growth pathways. This mechanistic complexity underscores a broader principle established in other pathophysiological contexts: GAS5’s function is fundamentally cell-type-specific and best resolved at the single-cell level. For instance, in the kidney, scRNA-seq revealed that GAS5 is ubiquitously expressed but highest in immune cells, such as NK/T lymphocytes. Its knockout led to specific shifts in cellular proportions and a complete rewiring of cell-cell communication networks. This included the turning off of immune regulatory pathways (CD45, CD6) and the turning on of pro-fibrotic pathways between fibroblasts and epithelial cells (Zhang X. et al., 2024). This paradigm suggests that in CRC, bulk downregulation of GAS5 likely masks complex, cell-type-specific expression changes and disrupted intercellular crosstalk within the TME that drive progression.

GAS5 also regulates the Hippo/YAP pathway in CRC. GAS5 directly binds the WW domain of YAP (Yes-associated protein), facilitating YAP phosphorylation and cytoplasmic retention. Phosphorylated YAP is then ubiquitinated and degraded, preventing its oncogenic nuclear function. In CRC models, this GAS5–YAP interaction significantly inhibited tumor cell proliferation; higher GAS5 correlated with lower YAP levels in patient tumors. Thus, GAS5 promotes ubiquitin-mediated degradation of YAP in CRC (Ni et al., 2019). Future studies employing integrated single-cell multi-omics will be critical to map this regulatory layer. This strategy can directly test if the downregulation of GAS5 within defined CRC subpopulations, such as stem-like or drug-tolerant cells, is spatially and functionally coupled to the hypermethylation and silencing of key tumor suppressor loci. Establishing this link would provide a mechanistic bridge between GAS5 expression, epigenetic reprogramming, and the emergence of therapy-resistant disease.

GAS5 may also impact p53 and cell cycle regulators. GAS5 upregulates its own intronic snoRNAs following DNA damage, which activates p53 signaling and cell cycle arrest in CRC cells. It can also scaffold transcription factors: GAS5 enhances E2F1 binding to the CDKN1B promoter, upregulating p27Kip1and inhibiting proliferation (Nguyen et al., 2025). These epigenetic-like roles demonstrate GAS5’s multifaceted regulation of CRC cell growth. A GAS5 promoter polymorphism (rs55829688 T>C) was linked to altered CRC risk by changing GAS5 transcription. Interestingly, most studies classify GAS5 as a tumor suppressor in CRC, but a few reports the opposite. For example, one Chinese study found upregulated GAS5 in CRC tissues and that GAS5 knockdown promoted malignancy (Wang et al., 2019), a finding at odds with others (Xie et al., 2022). This controversy underscores the critical need for single-cell and spatial resolution. Conflicting bulk results could arise from differences in the cellular composition of tumor samples. Spatial transcriptomics could resolve this by mapping GAS5 expression and help distinguish its levels in the tumor core, invasive front, and associated stroma.

Recent studies reveal that GAS5 is downregulated in T2DM and plays roles in insulin secretion and sensitivity. Serum GAS5 levels are significantly lower in T2DM patients than in healthy controls, correlating inversely with HbA1c and fasting glucose. In pancreatic β-cells, GAS5 overexpression increased glucose-stimulated insulin secretion and insulin content, whereas GAS5 knockdown impaired these functions. Mechanistically, GAS5 acts as a ceRNA, sequestering miR-29a-3p, miR-96-3p, and miR-208a-3p, which normally inhibit insulin signaling. By suppressing these miRNAs, GAS5 upregulates key insulin pathway components, including the insulin receptor (IR), insulin receptor substrate (IRS), and PI3K regulatory subunit, thereby amplifying insulin responsiveness. These findings highlight GAS5 as a critical regulator of β-cell function through a miRNA-dependent mechanism, suggesting its potential as a therapeutic target in T2DM (Luo et al., 2020). In the diabetic pancreas, scRNA-seq is needed to map GAS5 expression not only in β-cells but also in α-cells, δ-cells, ductal cells, and infiltrating immune cells. This could reveal if GAS5 loss in a specific endocrine subpopulation or in intra-islet macrophages drives dysfunction. Furthermore, spatial transcriptomics could determine whether GAS5 regulatory circuits differ between islets and peri-pancreatic adipose tissue depots, thereby influencing local insulin resistance.

In adipose tissue, GAS5 also promotes insulin sensitivity. Shi et al. (2019) demonstrated that GAS5 binds the promoter of the insulin receptor gene and upregulates IR expression; depletion of GAS5 in adipocytes impaired insulin signaling and glucose uptake. They identified a small molecule (NP-C86) that binds GAS5 and prevents its decay, thereby stabilizing GAS5 in diabetic adipocytes. Treatment with NP-C86 restored GAS5 levels and enhanced glucose uptake in patient-derived adipocytes. These findings indicate that GAS5 directly regulates insulin signaling genes in metabolic tissues (Shi et al., 2019). In summary, GAS5 acts as a metabolic regulator: GAS5 downregulation contributes to insulin resistance and β-cell dysfunction in diabetes, while its upregulation enhances insulin receptor signaling and glucose homeostasis (Shi et al., 2019; Luo et al., 2020). Several reviews highlight GAS5’s roles in diabetes pathogenesis and its potential as a biomarker or therapeutic target (Luo et al., 2020; Alharbi, 2024). GAS5 influences both CRC and diabetes via overlapping pathways. Notably, PI3K/AKT signaling is central in both diseases. In CRC, GAS5 suppresses PI3K/AKT by sponging miR-21, which otherwise inhibits PTEN (Nguyen et al., 2025; Xie et al., 2022). In diabetes, GAS5 boosts insulin signaling through IR/IRS/PI3K upregulation (Luo et al., 2020). Thus, GAS5 loss can potentiate AKT-driven proliferation in cancer and diminish AKT-mediated glucose uptake in diabetes.

GAS5’s suppression of growth-promoting pathways (Hippo/YAP, PI3K/AKT) in CRC contrasts with its enhancement of metabolic signaling (insulin pathway) in diabetes. However, these outcomes converge on controlling cell growth and survival under stress. For example, both YAP and insulin receptor are growth- and metabolism-related regulators. Interestingly, GAS5-mediated YAP degradation (via ubiquitination) in CRC (Ni et al., 2019) parallels GAS5-driven IR upregulation in adipocytes (Luo et al., 2020): one inhibits an oncogenic driver, the other boosts a metabolic receptor. GAS5 has emerged as a critical epigenetic modulator in both oncogenesis and metabolic disorders, exhibiting distinct yet mechanistically similar roles in CRC and T2DM. Our analysis of current evidence reveals that GAS5 orchestrates disease progression primarily through two interconnected epigenetic mechanisms: miRNA sponging and chromatin remodeling, particularly through interactions with EZH2-mediated histone modifications (Nguyen et al., 2025).

In CRC pathogenesis, GAS5 demonstrates potent tumor-suppressive functions through its ability to regulate key oncogenic pathways. The molecule serves as a ceRNA for multiple oncogenic miRNAs, most notably miR-182-5p, which is frequently overexpressed in CRC. By sequestering miR-182-5p, GAS5 derepresses critical tumor suppressor genes, including FOXO1 and SMAD4, thereby inhibiting proliferation and metastasis (Nguyen et al., 2025). This regulatory function parallels findings in atherosclerosis models where GAS5 modulates cholesterol metabolism through similar ceRNA mechanisms (Meng et al., 2020). Furthermore, GAS5 interacts with the epigenetic modifier EZH2, preventing H3K27me3-mediated silencing of tumor suppressor loci (Nguyen et al., 2025). Also, GAS5 facilitates E2F1 binding to the CDKN1B (p27) promoter, further suppressing tumor growth (Nguyen et al., 2025). This dual regulatory capacity positions GAS5 as a master regulator of CRC progression, with its downregulation contributing to disease pathogenesis through both transcriptional and post-transcriptional mechanisms.

The role of GAS5 in T2DM reveals striking mechanistic similarities to its function in cancer. In pancreatic β-cells, GAS5 maintains insulin secretion capacity by sponging miR-29a-3p and miR-96-3p, thereby preserving the expression of insulin signaling components, including INSR, IRS-1, and PIK3R1. The observed downregulation of GAS5 in diabetic patients correlates with impaired glucose homeostasis and reduced β-cell function, suggesting its potential as both a diagnostic marker and therapeutic target (Luo et al., 2020). Notably, the EZH2 mediated epigenetic silencing mechanism described in atherosclerosis models may also operate in metabolic tissues, though this requires further investigation in diabetes-specific contexts. Therapeutic strategies targeting GAS5 expression present exciting opportunities for both CRC and T2DM management. In CRC, restoration of GAS5 through lncRNA mimics or epigenetic modulators could simultaneously reactivate tumor suppressor pathways and inhibit oncogenic miRNA activity. For T2DM, enhancing GAS5 expression may improve β-cell function and insulin sensitivity through modulation of the miR-29a/PTEN/PI3K axis. However, the development of tissue-specific delivery systems will be crucial given GAS5’s context-dependent functions across different cell types.

GAS5 also engages ubiquitin‐mediated pathways. The best characterized case is YAP in CRC: GAS5 binding causes YAP phosphorylation and ubiquitin-dependent degradation (Ni et al., 2019). This highlights how GAS5 regulates protein stability of oncogenic factors. In diabetes models, GAS5 stability itself is controlled by the nonsense mediated decay factor UPF1 (Shi et al., 2019), which is not ubiquitin-based but is a regulated RNA decay process. Nonetheless, the YAP example exemplifies how GAS5 can link to the ubiquitin‐proteasome system to modulate signaling proteins. Given its dual roles, GAS5 is a potential therapeutic target in both CRC and diabetes, but approaches must be disease-specific. In CRC, strategies might aim to restore GAS5 expression or mimic its function. For example, nanoparticle‐delivered GAS5 or viral vectors could increase GAS5 in tumors, thereby reactivating p53 pathways and suppressing YAP and AKT signaling (Nguyen et al., 2025; Ni et al., 2019). Similarly, in diabetes, the therapeutic strategy involves leveraging GAS5 to alleviate repression of insulin signaling pathways, thereby restoring glucose homeostasis. This concept is exemplified by the small molecule NP-C86, which binds to GAS5, inhibits its degradation, and consequently enhances insulin sensitivity in diabetic adipocytes (Shi et al., 2019). Furthermore, GAS5 overexpression in β cells has been proposed as a potential approach to preserve insulin secretion (Luo et al., 2020).

## HNF1A-AS1

HNF1A-AS1 is a primate‐specific long non-coding RNA transcribed antisense to the HNF1A gene. It is broadly expressed in HNF1A-expressing tissues, pancreas, liver, and kidney, and localized predominantly in the nucleus (Beucher et al., 2022; Sepehri et al., 2022). Dysregulation of HNF1A-AS1 has been reported in many cancers, including GC, liver cancer, glioma, lung cancer, and CRC, often correlating with poor outcomes (Sepehri et al., 2022; Zhang Y. et al., 2022). In cancers such as CRC, liver, and lung cancer, HNF1A-AS1 typically acts as an oncogene: it is overexpressed in tumors, promotes proliferation, migration, and metastasis, and predicts worse survival (Zhang Y. et al., 2022; Fang et al., 2017).

Mechanistically, HNF1A-AS1 can function as a molecular scaffold or “sponge”: it binds chromatin regulators and miRNAs, thereby altering gene expression. For example, it binds the PRC2 component EZH2 to modulate histone methylation (Sepehri et al., 2022), and it scaffolds PRMT1 and the nuclear receptor PXR to induce histone methylation of target genes such as CYP3A4. HNF1A AS1 can also bind to the HNF1A protein itself, preventing its ubiquitination and degradation. Thus, HNF1A-AS1 integrates transcriptional and post-translational regulation, with pleiotropic effects on cell growth and metabolism (Wang et al., 2024).

The cell-type-specific expression and function of HNF1A-AS1, as revealed by single-cell studies across multiple tissues, suggest its impact is not uniform but concentrated in specific cellular compartments: pancreatic β cells (Bandesh et al., 2025; Bernardo et al., 2025), intestinal epithelial cells (Braun et al., 2023), GC stem cells (Zhao et al., 2024), and esophageal adenocarcinoma cells (Yang et al., 2014). This convergence on epithelial and endocrine cell types, precisely those responsible for hormone secretion, nutrient absorption, and barrier function, provides a molecular framework for understanding how its dysregulation could simultaneously contribute to metabolic dysfunction in diabetes and neoplastic transformation in colorectal cancer. In the pancreas, Bernardo et al. (2025) analyzed 2,036 human β cells and demonstrated that HNF1A-AS1 is among the top 20 genes most strongly correlated with HNF1A expression across individual β cells. This establishes that HNF1A-AS1 is co-expressed with HNF1A specifically within the β cell population at single-cell resolution and is an integral component of the HNF1A transcriptional program in native human islets.

Bandesh et al. (2025) provided the most comprehensive single-cell characterization of HNF1A-AS1 to date, profiling a dataset of 245,878 islet cells from 48 donors spanning the spectrum from non-diabetic to type 2 diabetic states. This analysis, comprising 99,029 β cells, identified HNF1A-AS1 as one of 511 robust differentially expressed genes in T2D versus non-diabetic β cells. Critically, HNF1A-AS1 expression alterations were specific to β cells, with minimal changes detected in α cells, δ cells, γ cells, or immune cell populations within the islet, establishing that this lncRNA operates within the insulin-producing β cell compartment itself. Braun et al. (2023) used single-cell RNA sequencing of human colon and ileum to demonstrate that HNF1A-AS1 is predominantly expressed in intestinal epithelial cells, with minimal detection in immune or stromal compartments. This epithelial-specific localization was further validated using isolated intestinal organoids compared to peripheral blood mononuclear cells, confirming that HNF1A-AS1 expression is enriched in the epithelial compartment. They also established the functional significance of HNF1A-AS1 in intestinal epithelium through the generation of intestine-specific HNF1A-AS1 knockout mice. These mice exhibited significantly increased susceptibility to DSS-induced colitis, demonstrating that HNF1A-AS1 functions within intestinal epithelial cells to maintain mucosal barrier integrity; a finding highly relevant to colitis-associated colorectal cancer (Braun et al., 2023).

This framework is supported by the finding that HNF1A-AS1 is consistently reduced in the inflamed intestinal epithelium of patients with ulcerative colitis and Crohn’s disease, with its downregulation correlating with disease severity (Braun et al., 2023). Chronic inflammation in inflammatory bowel disease is a well-established risk factor for colitis-associated colorectal cancer. Therefore, the loss of HNF1A-AS1 in this context may represent an early event in the inflammation-dysplasia-carcinoma sequence, directly linking epithelial dysfunction to the increased cancer risk observed in IBD patients. Furthermore, in research done by Zhao et al. (2024) on GC, they have demonstrated that HNF1A-AS1 is specifically enriched in GC stem cells, a subpopulation of tumor cells characterized by high aldehyde dehydrogenase activity, sphere-forming capacity, and expression of stemness markers OCT4, SOX2, and NANOG. Using flow cytometry to isolate ALDH-positive cells, they showed that HNF1A-AS1 expression is significantly higher in the stem-like compartment compared to bulk tumor cells, indicating that HNF1A-AS1 functions within the tumor epithelial compartment itself, specifically in the subset of cells responsible for tumor initiation and metastasis (Zhao et al., 2024).

In CRC, HNF1A-AS1 is upregulated in tumors versus normal mucosa, and high expression correlates with poor prognosis. Functionally, HNF1A-AS1 promotes CRC progression; its knockdown suppresses cell proliferation, invasion, and metastasis both *in vitro* and *in vivo*. Multiple molecular mechanisms underlie this. Notably, HNF1A-AS1 acts as a ceRNA for tumor-suppressive miRNAs. For instance, it sequesters miR-34a, thereby derepressing SIRT1; this interaction triggers p53 pathway inactivation and concurrently activates the canonical Wnt/β-catenin signaling. Through this miR-34a/SIRT1/p53 axis, HNF1A-AS1 promotes epithelial–mesenchymal transition and metastasis of CRC cells (Fang et al., 2017). In parallel, HNF1A-AS1 sponges miR-124, increasing expression of its target MYO6; this enhances CRC cell migration, invasion and glycolytic activity (Guo et al., 2020). A separate mechanism entails the transcriptional upregulation of angiogenic mediators, where HNF1A-AS1 recruits PBX3 to drive OTX1 expression. This cascade activates the ERK/MAPK pathway, ultimately fostering tumor angiogenesis (Wu et al., 2020).

HNF1A-AS1 exerts effects through post-translational and epigenetic mechanisms. On ubiquitination, HNF1A-AS1 can either inhibit or promote protein degradation depending on the context. In hepatocytes, HNF1A-AS1 binds the HNF1A protein and obstructs its E3 ubiquitin ligase TRIM25, preventing HNF1A ubiquitination and proteasomal degradation (Wang et al., 2024; Liu et al., 2018). This stabilizes HNF1A, enhancing its transcriptional program for metabolic genes. By contrast, in GC, HNF1A-AS1 drives the ubiquitination of the cell-cycle inhibitor p21. It upregulates the E3 ligase CDC34 and facilitates p21 ubiquitylation and degradation. Loss of p21 frees CDKs to drive proliferation (Liu et al., 2018). It also, in GC stem cells, HNF1A-AS1 acts as a ceRNA for miR-150-5p, upregulating β-catenin and activating Wnt signaling, with CMYC subsequently binding the HNF1A-AS1 promoter to create a positive feedback loop sustaining stemness. This work establishes that HNF1A-AS1’s oncogenic function is executed within cancer stem cells themselves through coordinated post-transcriptional and transcriptional mechanisms (Zhao et al., 2024). Furthermore, HNF1A-AS1 knockdown in esophageal adenocarcinoma cells preferentially affects genes involved in chromatin and nucleosome assembly, resulting in inhibited cell migration and invasion (Yang et al., 2014). These dual functions demonstrate that HNF1A-AS1 can reprogram ubiquitin-proteasome pathways, selectively stabilizing oncogenic drivers (such as HNF1A) while targeting tumor suppressors (such as p21) to exacerbate disease progression.

Epigenetically, HNF1A-AS1 regulates gene expression by interacting with chromatin-modifying enzymes. In CRC cells, it binds EZH2 (a component of the Polycomb Repressive Complex 2, PRC2) and forms nuclear RNA–DNA triplexes, potentially recruiting H3K27 methylation to silence tumor suppressor genes (Sepehri et al., 2022), Additionally, in GC, HNF1A-AS1 scaffolds PRMT1 with PXR to facilitate histone arginine methylation, activating target genes (Wang et al., 2024). Through these mechanisms, HNF1A-AS1 modulates DNA methylation and histone modifications, significantly altering gene expression programs. In summary, HNF1A-AS1 amplifies several oncogenic pathways in CRC, notably Wnt/β-catenin, PI3K/AKT, and MAPK, by acting as a molecular hub for miRNAs and transcriptional regulators (Fang et al., 2017; Wu et al., 2020; Liu et al., 2020).

While HNF1A-AS1 itself has not been extensively studied in diabetes, its host gene, HNF1A, is a well-known diabetes gene. HNF1A encodes a liver/pancreatic transcription factor critical for insulin secretion and glucose metabolism. Loss-of-function mutations in HNF1A cause MODY3 (a monogenic diabetes) and increase T2D risk, whereas gain-of-function variants in HNF1A protect against T2D (DeForest et al., 2023). HNF1A regulates many metabolic genes, including GLUT2, PKLR, insulin, and enzymes of glycosylation (Tudor et al., 2022). By analogy, HNF1A-AS1 is poised to modulate these processes. For instance, HNF1A-AS1 binds the HNF1A protein and prevents its degradation. This stabilization of HNF1A by HNF1A-AS1 could enhance HNF1A-driven transcription in β-cells and liver, potentially improving insulin production and glucose handling. Indeed, in hepatocytes, HNF1A-AS1 increases CYP3A4 expression by scaffolding HNF1A (Wang et al., 2024); similarly, it may amplify HNF1A’s regulation of metabolic genes.

Critically, genetic studies underline the link between HNF1A‐antisense transcription and diabetes. The HNF1A antisense promoter region (termed HASTER) produces HNF1A-AS1 transcripts in pancreatic β-cells and the liver. Experimental deletion of HASTER in mice leads to cell‐specific dysregulation of HNF1A: β-cells lose proper HNF1A expression, resulting in impaired insulin secretion and diabetes (Beucher et al., 2022). Thus, the HNF1A-AS1 locus is essential for maintaining HNF1A levels and glucose homeostasis.

Bernardo et al. further demonstrated that HNF1A-AS1 expression is dynamically regulated in β-cell subpopulations associated with T2D. Using lineage-specific Cre mice, they showed that HNF1A functions in a β-cell-autonomous manner to maintain glucose homeostasis, with liver- or gut-specific deletion failing to recapitulate the diabetic phenotype. In human islets, they identified distinct β cell clusters based on HNF1A activity: β1 cells with high HNF1A activity, and correspondingly high HNF1A-AS1 expression, and β2 cells with low HNF1A activity. Notably, individuals with T2D showed a marked inversion of these β cell states, with the β2/β1 cell ratio being approximately eight-fold higher in T2D versus non-diabetic donors (Bernardo et al., 2025). This indicates that HNF1A-AS1 downregulation is a feature of the β-cell state associated with diabetes. Complementing this, Bandesh and colleagues integrated single-cell data with T2D genetics, revealing that HNF1A-AS1 is among eight genes linked to independent T2D association signals, nominating it as a candidate causal gene in T2D pathophysiology (Bandesh et al., 2025). In humans, variants at the HNF1A locus (including in antisense regions) are associated with glycosylation patterns and inflammation (Tudor et al., 2022) pathways that intersect with both diabetes and cancer. Collectively, these data suggest that HNF1A-AS1 could influence diabetes risk by modulating HNF1A activity and related metabolic networks. For example, HNF1A-AS1-mediated enhancement of HNF1A stability via blocking its ubiquitination (Wang et al., 2024) could increase expression of insulin and glucose transport genes, whereas loss of HNF1A-AS1 (or HASTER activity) impairs β-cell function (Beucher et al., 2022).

Both CRC and diabetes exhibit metabolic dysregulation, with HNF1A-AS1 playing a central role in shared pathways. A key intersection is cellular metabolism. HNF1A-AS1 drives aerobic glycolysis (the “Warburg effect”) in cancer cells (Guo et al., 2020; Cheng et al., 2022), mirroring the metabolic shifts seen in diabetes. Under chronic high glucose conditions, HNF1A-AS1 further amplifies glycolytic activity by sponging miR-124 and upregulating MYO6 (Guo et al., 2020; Cheng et al., 2022). This mechanism directly ties CRC’s metabolic reprogramming to the hyperglycemic environment of diabetes, highlighting HNF1A-AS1 as a critical node linking these diseases. PI3K/AKT signaling is another common axis: HNF1A-AS1 activates PI3K/AKT in gastric and likely CRC cells by sponging miR-30b-3p, leading to increased PIK3CD expression (Liu et al., 2020). This pathway, PI3K/AKT, is critically involved in both insulin receptor signaling and cancer cell survival (Huang et al., 2018). Similarly, HNF1A-AS1 upregulates Wnt/β-catenin signaling through the miR-34a/SIRT1 axis (Fang et al., 2017), a pathway important for both β-cell function and intestinal epithelial homeostasis. Additional shared pathways include: ERK/MAPK activation via OTX1 in CRC (Wu et al., 2020), which may interact with insulin/IGF-1 signaling. Moreover, HNF1A-AS1 influences glycosylation and inflammation: HNF1A regulates fucosylation enzymes, and polymorphisms in HNF1A-AS1 are linked to plasma N-glycan change, a phenomenon seen in diabetes-related inflammation (Tudor et al., 2022).

Beyond its role in cancer, HNF1A also influences metabolic and glycosylation pathways. It regulates genes involved in fucosylation, glucose metabolism, and insulin. Environmental factors can modify HNF1A’s DNA methylation patterns, leading to changes in plasma protein glycosylation. Notably, methylation at four specific CpG sites in HNF1A’s first exon affects RNA transcript levels and alters branched N-glycan structures. Targeted demethylation of these sites reduces complex, core-fucosylated N-glycans, demonstrating that epigenetic regulation of HNF1A directly impacts its expression and glycosylation functions (Tudor et al., 2022). These findings position HNF1A-AS1 as a central regulator of growth factor signaling and metabolic networks common to both CRC and diabetes. Through its effects on insulin-responsive pathways (PI3K/AKT, MAPK), cellular metabolism (glycolysis), and stress responses (p53/SIRT1, glycosylation), HNF1A-AS1 may promote tumorigenesis in patients with diabetes. This provides a plausible molecular explanation for the observed increased CRC risk in diabetes, which may be mediated through hyperinsulinemia, chronic inflammation, and HNF1A-AS1-dependent alterations in cell signaling. This mechanistic link establishes a critical molecular bridge between chronic inflammation and cancer predisposition, holding particular significance for the T2D-CRC axis given that the pro-inflammatory milieu is a hallmark of T2D. Although existing studies have provided critical single-cell resolution data on HNF1A-AS1 in pancreatic β cells and intestinal epithelium, spatial transcriptomics mapping its expression across tissue architecture—specifically the colon crypt-villus axis, pancreatic islet microenvironment, and the epithelial-to-carcinoma transition—remains unexplored. Such spatial insights would significantly elucidate the functional roles of HNF1A-AS1 within the T2D and CRC tissue microenvironments, marking a critical avenue for future investigation.

## H19

H19 is a multifunctional maternal lncRNA involved in cell growth and epigenetic regulation, located on Chromosome 11 with a shared locus with IGF2. H19 is also involved in metabolic diseases and many cancers, including CRC. It also plays a role in insulin resistance and inflammation, and although increased in CRC, it has a positive correlation in Non-diabetic CRC patients, while a negative correlation in diabetic CRC cases, by 1% different from non-diabetic CRCs, which indicates its value as a therapeutic and diagnostic factor (Zhao M. et al., 2021; Hernández-Aguilar et al., 2021; Liao et al., 2023; Rajendran et al., 2024). The long non-coding RNA H19 drives colorectal carcinogenesis through multiple pathways, exerting both oncogenic and context-dependent tumor-suppressive effects. As an oncogene, H19 promotes tumor growth by binding to the translation initiation factor eIF4A3, elevating CDK4, Cyclin D1, and Cyclin E1 levels, thereby accelerating cell cycle progression and proliferation (Zhao M. et al., 2021). Additionally, H19 facilitates metastasis by modulating the Wnt/β-catenin and Ras/MAPK pathways. Through the H19/miR-29 b-3p/PGRN axis, it inhibits miR-29 b-3p, derepressing PGRN to activate Wnt signaling and induce EMT, downregulating E-cadherin while upregulating Vimentin and SNAI1. Concurrently, H19 activates the Ras/RAF/MEK/ERK cascade, enhancing migration and invasion in CRC cells (Chowdhury et al., 2023). Single-cell RNA sequencing in colorectal cancer has confirmed that H19 is significantly overexpressed in tumor epithelial cells compared to normal epithelial cells, and CRISPR/Cas9 knockout experiments demonstrated that H19 is functionally essential for CRC cell survival, as H19 knockout in HCT116 cells resulted in decreased cell viability (Li et al., 2025). Furthermore, analysis of the essential long non-coding RNA (ESL) gene set, which includes H19 alongside 28 other lncRNAs, revealed enrichment not only in tumor epithelial cells but also in immune subsets such as T cells and B cells (Li et al., 2025).

H19 plays a crucial role in CRC progression by modulating epigenetic mechanisms, including histone modifications, DNA methylation, and miRNA sponging, collectively influencing gene expression and tumor behavior (Liao et al., 2023; Chowdhury et al., 2023; Chen et al., 2019; Farooqi et al., 2022; Li et al., 2016; Lim et al., 2021). One key mechanism involves HNRNPA2B1, an RNA-binding protein and m6A reader, which interacts with H19 to amplify its oncogenic effects. H19 binds HNRNPA2B1, stabilizing RAF-1 mRNA and activating the RAF-ERK signaling pathway. Phosphorylated ERK further upregulates SNAI1, a key EMT transcription factor, driving CRC invasion. Additionally, HNRNPA2B1 promotes colon cancer proliferation by activating the ERK/MAPK pathway, which is frequently upregulated in clinical CRC specimens. This suggests that targeting the H19/HNRNPA2B1/RAF-ERK axis could be a promising therapeutic strategy (Chowdhury et al., 2023; Tang et al., 2021).

Beyond histone modifications, H19 exerts epigenetic control through interactions with Polycomb Repressive Complex 2 (PRC2). In bladder cancer, nuclear H19 binds EZH2, the catalytic subunit of PRC2, to silence tumor suppressor genes, facilitating metastasis. Similarly, H19 recruits MBD1 (Methyl-CpG-Binding Domain Protein 1) to differentially methylated regions (DMRs), repressing imprinted genes critical for growth regulation. H19 also encodes miR-675, which fine-tunes gene expression post-transcriptionally by targeting IGF1R, impacting cell proliferation and differentiation (Gao et al., 2014). H19 exhibits dual roles in cancer progression through its function as a ceRNA. By sponging miR-200a and miR-138, H19 upregulates SIRT1, which deacetylates p53, RB1, and E2F1, thereby activating oncogenic pathways including Rb/E2F and CDK8/β-catenin. Paradoxically, H19 can also stabilize p53 and enhance the apoptotic effects of the HDAC inhibitor ITF2357 (givinostat). This contradictory behavior highlights the context-dependent nature of H19’s role in cancer development, demonstrating both tumor-promoting and tumor-suppressing functions (Farooqi et al., 2022; Yang et al., 2021; Zichittella et al., 2023).

Beyond its functions in tumor cells, H19 operates within immune cell populations to shape an immunosuppressive microenvironment. In pancreatic cancer, H19 in tumor-associated macrophages (TAMs) promotes their polarization toward the immunosuppressive M2 phenotype through a distinct mechanism: H19 competitively binds YTHDC1 mRNA with miR-107 and interacts with YTHDC1 protein, regulating SRSF1 stability and affecting alternative splicing of IL-6 and IL-10 (Liu P. et al., 2025). Bioinformatic analysis across multiple cancer types, including thyroid carcinoma, has revealed that H19 expression correlates with infiltration levels of diverse immune cells, including CD4^+^ T cells, CD8^+^ T cells, B cells, dendritic cells, neutrophils, and macrophages, suggesting that H19-immune interactions are conserved across tumor types (Sahin, 2023). However, the direction of H19 expression varies by cancer type; downregulated in thyroid carcinoma versus upregulated in CRC and most other cancers, emphasizing the tissue-specific and context-dependent nature of H19 biology (Li et al., 2025; Sahin, 2023).

Adding another layer of complexity, a paradigm-shifting discovery has revealed that H19 encodes a previously undetected protein, H19-IRP (H19-immune-related protein), which functions distinctly from H19 RNA. Using scRNA-seq of 17 glioblastoma samples, researchers found that H19 is expressed exclusively in tumor cells, and H19-IRP localizes to the nucleus where it acts as a transcription factor for CCL2 and Galectin-9, leading to their upregulation. This transcriptional activation recruits myeloid-derived suppressor cells (MDSCs) and TAMs while promoting T cell exhaustion, thereby shaping an immunosuppressive tumor microenvironment (Chen J. et al., 2024). Elegant rescue experiments demonstrated that H19 RNA promotes tumor cell proliferation and migration, whereas H19-IRP specifically mediates immunosuppression, revealing that H19’s dual functionality arises from two distinct molecular entities encoded by the same gene (Chen J. et al., 2024). In glioblastoma, the H19-IRP acts as a tumor-associated antigen displayed on MHC-I molecules, highlighting its potential as a promising target for cancer immunotherapy. A circular RNA vaccine targeting H19-IRP demonstrated potent anti-tumor efficacy in preclinical models, remodeling the tumor microenvironment by reducing MDSCs and TAMs while increasing CD8^+^ and CD4^+^ T cell infiltration and enhancing T cell cytotoxicity. This vaccine strategy, when combined with immune checkpoint blockade, elicited synergistic effects, indicating that targeting H19-IRP has the potential to bypass resistance to standard immunotherapies (Chen J. et al., 2024).

This intricate regulation extends beyond cancer into metabolic physiology. In diabetic conditions, the hyperinsulinemia leads to disruption of PI3K/AKT, which leads to reduced H19 levels and subsequently increased let-7, suppressing metabolic genes like Insr and Lpl, impairing glucose regulation. Conversely, in healthy muscle, insulin spikes trigger let-7 to degrade H19, forming a protective feedback loop that prevents excessive glucose uptake and maintains metabolic balance. Notably, insulin administration was shown to suppress H19 expression through a PI3K/AKT-dependent pathway involving phosphorylation of the RNA-binding protein KSRP, which promotes let-7 maturation while simultaneously destabilizing H19 transcripts. The H19/let-7-PI3K/Akt interplay reveals a dynamic checkpoint in insulin signaling, offering new mechanistic insights into metabolic disease and potential therapeutic strategies (Zhao M. et al., 2021; Nie et al., 2016).

High glucose directly regulates H19 via epigenetic mechanisms; specifically, studies in HTR8/SVneo trophoblast cells revealed that elevated glucose levels significantly upregulate H19 by modifying DNA methylation at 12 CpG sites within its promoter. Functional validation confirmed this mechanism, as methylated H19 promoter plasmids exhibited significantly higher luciferase activity compared to unmethylated controls (Zhang Q. et al., 2022). This provides a direct mechanism linking hyperglycemia to epigenetic dysregulation of H19 in epithelial cells, with implications for both diabetic complications and cancer. Studies in diabetic peripheral artery disease patients utilized gene enrichment analysis to implicate H19 in IGF2BP pathways, establishing a functional link to glucose metabolism and vascular smooth muscle activity. Since IGF2BPs are also critical for cell migration and stem cell renewal (Yundung et al., 2024), they likely reinforce the interplay between diabetes, insulin-like growth factors, and cancer risk. Furthermore, multi-omics data from colorectal cancer reveal that lncRNAs such as PVT1 are subject to epigenetic regulation where CpG hypomethylation drives increased expression. This evidence strongly suggests that H19 may similarly be governed by hypomethylation-mediated transcriptional activation (Li et al., 2025). In hyperinsulinemia,H19 increases DNMT3 expression, epigenetically altering the DMRs of H19/IGF2, offering H19 as a promising therapy for diabetes control (Liao et al., 2023).

The complexity of H19 epigenetic regulation is further highlighted in embryogenesis, where maternal obesity disrupts H19/IGF2 imprinting by altering DNA methylation at the imprinting control region (ICR). This disruption results in biallelic H19 expression and a concurrent downregulation of IGF2. By integrating single-cell RNA sequencing with GeoMx spatial transcriptomics, this study identified H19 expression in myogenic progenitors and differentiated myocytes. The findings revealed an antagonistic relationship between H19 and its mature product, miR-675: while H19 accumulation inhibits myogenesis, miR-675 promotes it. This delicate regulatory balance is critically disrupted under conditions of metabolic stress (Gao et al., 2025). This spatial resolution of H19 expression within specific cellular compartments provides a framework for understanding how its distribution within the tissue microenvironment influences both developmental processes and disease pathogenesis.

In CRC, hypoxia stabilizes HIF1α by inhibiting its VHL-mediated ubiquitination and degradation. This stabilized HIF1α transcriptionally upregulates H19, creating a critical axis that promotes tumor progression through enhanced proliferation, stemness, and chemoresistance. The HIF1α-H19 axis further influences cancer development by modulating the ubiquitination and stability of key oncoproteins and tumor suppressors (Liao et al., 2023; Zeng et al., 2022; Mu et al., 2023; Groulx and Lee, 2002). HIF1α orchestrates the ubiquitin-dependent regulation of several critical cancer-related proteins. It promotes tumor progression by facilitating the degradation of tumor suppressors, including MEN1, through the SCFFBXL1 E3 ligase complex. Also, HIF1α maintains its protein levels through USP51-mediated deubiquitination. USP51 binds to Elongin C (ELOC), disrupting the VHL ubiquitin ligase complex and preventing HIF1α ubiquitination. This dual regulation demonstrates how HIF1α eliminates tumor suppressors while preserving oncogenic proteins through coordinated ubiquitination control in CRC. The HIF1α-H19 axis thus represents a critical mechanism of hypoxia adaptation in CRC progression (Zeng et al., 2022; Mu et al., 2023). Throughout these diverse contexts, H19 exhibits opposing functions depending on cellular conditions; while it typically promotes tumorigenesis, under stress like hypoxia, it can suppress proliferation by inhibiting IGF1R or through its encoded miR-675. Furthermore, H19 and p53 antagonize each other; p53 represses H19 to curb tumor growth, whereas H19 overexpression can override p53-mediated suppression. Hypoxia-induced HIF1-α further complicates this dynamic by upregulating H19, which then silences tumor suppressors like cyclin-dependent kinase (CDK) inhibitors while activating oncogenes. Thus, although H19 knockdown generally inhibits cancer, its pleiotropic functions, shaped by cell type, microenvironmental stress, and genetic context, underscore the need for precisely targeted therapeutic strategies (Liao et al., 2023; Lim et al., 2021). Thus, although H19 knockdown typically inhibits cancer, its dual role, dependent on cell type and stress signals, highlights the need for context-specific therapeutic approaches.

The convergence of hypoxia signaling, epigenetic regulation, glucose metabolism, and immune modulation through H19 highlights its central role as a molecular hub integrating metabolic stress signals with cancer progression pathways. The integration of single-cell and spatial transcriptomics has been instrumental in elucidating H19’s cell-type specific expression patterns, predominantly in tumor epithelial cells, but also in immune subsets including T cells, B cells, and TAMs. These findings highlight the importance of developing context-specific, cell-type targeted therapeutic strategies that account for H19’s multifunctional nature, particularly given its opposing roles in diabetic versus non-diabetic CRC patients and its dual functions as both a non-coding RNA and a protein-encoding transcript.

## ANRIL

ANRIL (CDKN2B-AS1) is an lncRNA transcribed from the INK4b‐ARF‐INK4a gene cluster, a locus encoding three tumor suppressors with CDK inhibitor activity. Spanning approximately 126 kbp, ANRIL is located on chromosome 9p21 and is transcribed in the opposite direction to the protein-coding genes in this cluster. ANRIL plays a significant role in the pathogenesis of multiple cancers, including CRC, cholangiocarcinoma, breast cancer, and prostate cancer, among others. Its involvement in these diseases stems from its regulatory functions in cell proliferation, apoptosis, and epigenetic modulation (Huang et al., 2020; Mehta-Mujoo et al., 2019; Kong et al., 2018).

A critical aspect of ANRIL’s function is its cell-type-specific activity within the tumor microenvironment. While ANRIL is a well-established oncogenic driver in tumor cells themselves, emerging evidence highlights its role in stromal compartments. For instance, in non-small cell lung cancer (NSCLC), ANRIL is highly expressed in cancer-associated fibroblasts (CAFs), where it is packaged into exosomes and transferred to tumor cells, subsequently driving glycolytic metabolism and proliferation (Zhang B. et al., 2025). This indicates that ANRIL can operate in both fibroblasts and epithelial cancer cells, facilitating critical crosstalk within the tumor niche. This stromal-to-tumor cell communication adds a layer of complexity to its role in CRC progression, suggesting that the cellular origin of ANRIL is as important as its final destination.

ANRIL’s upregulation plays a critical role in both CRC progression and diabetes-related complications through the dysregulation of multiple signaling pathways. In the context of diabetes, ANRIL drives cardiac pathologies and metabolic dysfunction by regulating a diverse network of signaling cascades. These include the AGE-RAGE axis, the PI3K-AKT pathway, and key inflammatory mediators such as TNF-α, NF-κB, and IL-6. Furthermore, ANRIL modulates growth factor signaling, notably VEGF-C—a critical promoter of tumor metastasis—and the TGF-β1/Smad pathway. A significant overlap exists between cancer and diabetes, characterized by shared signaling hubs such as PI3K-AKT and VEGF-C, alongside oncogenic pathways including Wnt, NOTCH (Bonegio and Susztak, 2012), STAT3/NF-Κb (Mangum et al., 2024), MAPK/ERK (Zhang W. et al., 2011), and interactions with Let-7 miRNA. This convergence highlights the dual role of ANRIL in both pathologies. Additionally, ANRIL influences cancer progression by activating NF-κB via the linear ANRIL-p14AS and UBE2D3/IκBα signaling axis. It also disrupts the tumor-suppressive function of p53, further promoting tumorigenesis (Mehta-Mujoo et al., 2019; Sanchez et al., 2023; Sooshtari et al., 2022; Ma and Hu, 2023; Huang B. et al., 2023).

In the context of T2D, the functional relevance of ANRIL is most pronounced in pancreatic β-cells. Genetic analyses have identified that T2D-associated single-nucleotide polymorphisms (SNPs) at the ANRIL locus, specifically rs564398 in exon 2, are causally linked to diminished β-cell proliferation. This suggests that within the pancreatic islet, ANRIL operates primarily in the endocrine β-cell compartment to influence cell mass, a key determinant of insulin secretion capacity and diabetes susceptibility (López–Noriega and Rutter, 2021). The expression landscape of ANRIL in islets is further complicated by the presence of multiple circular isoforms (circANRIL), which are predominantly cytoplasmic and whose ratio to linear ANRIL is associated with β-cell proliferation and diabetes risk genotype (MacMillan et al., 2022).

Under hyperglycemic conditions, ANRIL accelerates CRC progression by sequestering miR-186-5p, a critical negative regulator of the oncogene HIF-1α. This sponging effect derepresses HIF-1α, driving oncogenic activity; notably, this dysregulation correlates with poor clinical prognosis in CRC patients characterized by high ANRIL and low miR-186-5p expression (Mosaad et al., 2024). Furthermore, ANRIL participates in a feedback loop with the RAS signaling pathway. RAS activation induces RREB1, which binds to the ANRIL promoter and represses ANRIL transcription. This reduction in ANRIL levels subsequently alleviates its inhibitory effect on CDKN2B, leading to increased CDKN2B expression. Since RAS signaling is a major driver of carcinogenesis, this regulatory interplay underscores ANRIL’s pivotal role in cancer development (Congrains et al., 2013).

ANRIL exerts its oncogenic and metabolic effects primarily through epigenetic silencing and post-transcriptional regulation. Functioning as a pivotal modulator of the Polycomb Repressive Complex 2 (PRC2)—a methyl transferase complex comprising EZH2, EED, and SUZ12—ANRIL facilitates the recruitment of EZH2, the complex’s catalytic subunit. This interaction drives the dimethylation and trimethylation of histone H3 at lysine 27 (H3K27me3), resulting in the transcriptional silencing of critical tumor suppressors, including CDKN2B and CDKN2A. This repressive state is further consolidated by ANRIL’s recruitment of CBX7 (a PRC1 component), which catalyzes the ubiquitination of histone H2A at lysine 119 (H2AK119ub).

Concurrently, ANRIL recruits DNA methyl transferase T3B (DNMT3B) to synergistically enforce a robust transcriptional silencing at the CDKN2A/B locus. This specific epigenetic hallmark is detected in approximately 40% of human cancers, spanning gastrointestinal, ovarian, prostate, and lung malignancies (Kong et al., 2018; Ma and Hu, 2023; Congrains et al., 2013).

Upon DNA damage, E2F1 transcriptionally activates ANRIL, which then recruits PRC2 to silence the CDKN2A/B locus. This action effectively suppresses the expression of the tumor suppressor p14^ARF^, thereby mitigating the DNA damage response. This mechanism impairs p53 stabilization by bypassing the inhibitory action of p14^ARF^ on MDM2. Concurrently, it enhances CDK4/6-cyclin D activity, leading to Rb phosphorylation and the subsequent liberation of E2F1. This feed-forward loop amplifies ANRIL expression, disrupts p53-mediated cell cycle arrest, and drives uncontrolled proliferation, a hallmark of cancer (Sanchez et al., 2023). Additionally, ANRIL binds to SOX2, enhancing WNT/β-catenin signaling to further accelerate tumor progression (Kong et al., 2018).

Beyond oncology, ANRIL plays a critical role in T2D via both genetic and epigenetic pathways. Multiple T2D-associated SNPs located near the ANRIL locus modulate diabetes susceptibility by directly impairing pancreatic islet function, an effect that occurs independently of the influence of adjacent protein-coding genes. Certain SNPs exhibit age-dependent regulation of ANRIL, either upregulating or downregulating its expression. This dysregulation may impair insulin secretion and compromise β proliferation. A critical risk SNP located in ANRIL exon 2 abolishes a specific methylation site. This epigenetic alteration disrupts the glucose-responsive proliferation of β-cells, thereby impairing their growth. Furthermore, high glucose conditions drive ANRIL upregulation, which in turn enhances VEGF expression through a PRC2-miR-200b axis. This molecular cascade establishes a direct link between ANRIL and DR. (Kong et al., 2018). ANRIL also contributes to diabetic microvascular complications, particularly DR. Under high glucose conditions, ANRIL is upregulated and promotes VEGF expression by recruiting PRC2 (EZH2) and suppressing miR-200b, an inhibitor of VEGF. In diabetic mice and human retinal cells, ANRIL silencing blocked VEGF-induced vascular leakage and angiogenesis, confirming its role in DR pathogenesis. Mechanistically, ANRIL binds p300 and EZH2, forming an epigenetic complex that activates VEGF, highlighting its potential as a therapeutic target for diabetic complications (Kong et al., 2018; Thomas et al., 2017).

Despite these advances, a significant gap remains in our understanding of the precise spatial distribution of ANRIL within complex tissues. Current knowledge is largely derived from bulk tissue analysis or *in vitro* cell models. Future studies employing spatial transcriptomics are urgently needed to map the expression of ANRIL and its isoforms within the intricate architecture of the colorectal tumor microenvironment and the pancreatic islet. Such spatially-resolved data would clarify whether ANRIL-driven crosstalk between CAFs and cancer cells, or between different islet endocrine cell types, occurs in specific niches, providing a clearer picture of the molecular interactions that drive T2D and CRC.

## Clinical chemistry implications of long non-coding RNAs in colorectal cancer and diabetes

LncRNAs have emerged as critical regulators of cellular signaling, metabolism, and epigenetic landscapes in both CRC and diabetes, and they hold significant potential as clinical chemistry biomarkers for diagnosis, prognosis, and therapeutic monitoring due to their stability in biofluids, tissue-specific expression, and involvement in disease-relevant pathways (Table 3). Many lncRNAs exhibit differential expression patterns in CRC and diabetes, which can be harnessed for early detection. For example, MIR31HG levels are elevated in CRC tissues and serum, correlating with a poor prognosis, tumor aggressiveness, and chemoresistance (Wang et al., 2022; Tu et al., 2020). In diabetic patients, circulating MIR31HG may reflect tissue hypoxia and inflammatory status (Gupta et al., 2020). Similarly, ANRIL expression correlates with CRC progression and diabetic complications such as retinopathy, and specific ANRIL SNPs and epigenetic modifications can stratify patients at risk for T2D and cardiovascular complications (Thomas et al., 2017; Zhu et al., 2022; Flood et al., 2020; Liu et al., 2022). KCNQ1OT1 is detectable in plasma and is associated with chemoresistance, tumor metastasis, and cardiac or renal dysfunction in diabetic patients (Liu et al., 2024; Yang et al., 2018b; Zhao et al., 2021b; Yang et al., 2018a). In contrast, GAS5 levels inversely correlate with CRC proliferation and insulin resistance, suggesting that serum GAS5 may be a potential dual biomarker for metabolic dysfunction and tumour suppression (Alharbi, 2024; Xie et al., 2022). HNF1A-AS1 and UCA1 are detectable in tissues and circulating exosomes, providing minimally invasive options for monitoring disease progression (Luan et al., 2020; Mohamadnejad et al., 2025; Bernardo et al., 2025; Yuan et al., 2022). UCA1 plasma levels differ between CRC patients and healthy controls, reflecting diabetes-associated metabolic stress.

**TABLE 3:** Clinical relevance and translational potential of key lncRNAs as biomarkers in type 2 diabetes and colorectal cancer.

LncRNA	Disease context	Clinical relevance	Sample source	Mechanistic link to clinical chemistry assays	Ref.
MIR31HG	CRC/Diabetes	Prognostic marker, tumor aggressiveness, hypoxia	Tissue, serum	Correlates with tumor stage, chemoresistance, HIF-1α, glycolysis	Cui et al. (2019), Huang et al. (2023a), Aghaei-Zarch (2024)
ANRIL	CRC/T2D complications	Risk stratification, diabetic retinopathy	Tissue, plasma	NF-κB-mediated inflammation, VEGF, epigenetic regulation	Huang et al. (2018), Lim et al. (2021), Gao et al. (2014)
KCNQ1OT1	CRC/Diabetic cardiomyopathy, nephropathy	Chemoresistance, fibrosis, metabolic dysregulation	Plasma, tissue	PI3K/AKT, TGF-β, NF-κB, glucose metabolism	Pan et al. (2021), Wang et al., 2017; Sahin (2023)
GAS5	CRC/T2D	Tumor suppressor, insulin sensitivity	Serum, tissue	PI3K/AKT signaling, IR/IRS regulation, Hippo/YAP, ubiquitination	Bai et al. (2023), Xian and Zhao (2019), Yundung et al. (2024)
HNF1A-AS1	CRC/Glucose metabolism	Prognostic marker, β-cell function, glycosylation	Tissue, exosomes	PI3K/AKT, MAPK, glycolysis, HNF1A stabilization	Yang et al. (2018a), Ramasubbu and Devi Rajeswari (2023), Luo et al. (2020)
UCA1	CRC/T2D	Chemoresistance, metabolic stress, angiogenesis	Tissue, plasma	miR-143/FGF21 axis, mTOR/STAT3, VEGF-C, NF-κB, epigenetic control	Luo et al. (2023), Craig-Schapiro et al. (2025), Neve et al. (2018), Luan et al. (2020)
H19	CRC/T2D	Oncogenic driver; insulin signaling regulator; epigenetic biomarker	Tissue, plasma, exosomes	Influences PI3K/AKT insulin signaling (glucose, insulin, HbA1c); Wnt/β-catenin tumor markers; DNMT3/EZH2 epigenetic signatures; hypoxia/HIF-1α-related VEGF and lactate changes	Alharbi (2024), Beucher et al. (2022), Wang et al. (2024)
MALAT1	CRC/Diabetes (T2D + complications)	Prognostic marker; predicts metastasis, chemoresistance; inflammation and insulin-resistance biomarker	Tissue, plasma, serum, endothelial cells, retina, kidney, liver, myocardium	Regulates Wnt/β-catenin, PI3K/AKT/mTOR, NF-κB, VEGF, PRC2-mediated epigenetics, alternative splicing; modulates ubiquitination (FOXP3, SREBP-1c)	Abdel-Nasser et al. (2023), Huang et al. (2022), Tiansheng et al. (2020), Chow and Poon (2013), Vana et al. (2007)

LncRNAs could complement traditional clinical chemistry markers, such as CEA and CA19-9 for CRC, or HbA1c, fasting glucose, and insulin resistance indices for diabetes. Combined measurement of MIR31HG or UCA1 with HbA1c could provide early risk stratification for CRC in diabetic patients. In contrast, quantification of GAS5 or ANRIL could identify individuals with both metabolic dysregulation and early neoplastic changes. Circulating exosomal lncRNAs enable repeated, minimally invasive sampling, facilitating the longitudinal monitoring of therapeutic response and glycemic control (Luan et al., 2020; Muto et al., 2022). Beyond diagnosis, lncRNAs also predict therapeutic efficacy. MIR31HG, KCNQ1OT1, and UCA1 levels correlate with chemoresistance in CRC, making them potential markers for adjusting chemotherapy regimens (Zhang et al., 2020; Wong et al., 2019; Liu S.-J. et al., 2021). GAS5 restoration strategies or small molecules that stabilize GAS5, such as NP-C86, may be evaluated in clinical studies by monitoring circulating GAS5 levels (Yundung et al., 2024). ANRIL and HNF1A-AS1 can serve as surrogate markers for pathway-targeted interventions, such as VEGF or PI3K/AKT inhibitors, especially in patients with comorbid diabetes (Guan et al., 2025; Guo et al., 2021; Muto et al., 2022).

The clinical chemistry significance of these lncRNAs stems from their regulation of key metabolic, inflammatory, and angiogenic pathways, which conventional assays can measure. In glucose metabolism and glycolysis, MIR31HG, UCA1, HNF1A-AS1, and KCNQ1OT1 regulate glycolysis, insulin signaling, and energy balance, thereby influencing fasting glucose, HbA1c, and lactate levels (Li et al., 2014; Cui et al., 2019; Huang et al., 2017; Cagle et al., 2021). For inflammatory markers, ANRIL, KCNQ1OT1, and UCA1 influence NF-κB and SASP-mediated inflammation, correlating with serum levels of CRP, IL-6, and TNF-α (Guan et al., 2025; Osmond et al., 2022; Craig-Schapiro et al., 2025; Maghool et al., 2026). Angiogenic markers—including VEGF and HIF-1α, which are modulated by MIR31HG, ANRIL, and UCA1—can be quantified in tandem with circulating lncRNAs. This combined approach enables the assessment of vascular risk in diabetes and tumor angiogenesis in CRC (Bonegio and Susztak, 2012).

Incorporation of lncRNAs into routine clinical chemistry platforms will require standardized methods for isolation, quantification, and normalization. Multiplex panels combining lncRNAs with protein or metabolite markers may provide integrated risk scores for patients with overlapping CRC and diabetes phenotypes. Exosome-based assays, qPCR, and next-generation sequencing are emerging as feasible approaches for clinical laboratories (Luan et al., 2020; Yan et al., 2021). Overall, lncRNAs represent a bridge between molecular biology and clinical chemistry, offering tools to diagnose, monitor, and predict therapeutic outcomes in CRC and diabetes. Their dual role in oncogenesis and metabolic regulation underscores their potential for personalized medicine, particularly in patients with comorbid conditions where shared molecular pathways exacerbate disease progression.

## Future perspectives

While this review emphasizes the role of lncRNAs as molecular links between T2D and CRC, recent studies highlight a more complex regulatory network involving both lncRNAs and miRNAs that govern systemic pathophysiology. Integrating this emerging literature broadens the scope of this review in three critical dimensions. First, it facilitates the integration of cardiovascular pathology into the metabolic-oncologic framework; since T2D patients face dual risks of oncogenesis and cardiovascular complications, novel insights reveal that miR-122 and miR-217 function as key regulators of tissue injury in myocardial infarction (MI) (Zaafan and Abdelhamid, 2025; Abdel-Nasser et al., 2023). Specifically, the miR-122/Sirt-6/ACE2 axis regulates oxidative stress, inflammation, and metabolic stability in the heart, providing a molecular basis for linking ncRNA dysregulation across cardiac, metabolic, and oncogenic pathways, while miR-217 serves as a promising diagnostic biomarker that, when quantified alongside lncRNA markers, could enhance early risk stratification for diabetic patients with multimorbidity. Second, it strengthens the understanding of the lncRNA/miRNA axis in cancer progression; although lncRNAs like H19 and MALAT1 are central to our analysis, studies such as Ayeldeen et al. (Ayeldeen et al., 2024) demonstrate the critical role of the NBAT1/miR-21 axis in CRC progression, and polymorphisms in PVT-1 significantly influence miR-145 expression levels, underscoring the need for comprehensive diagnostic models that account for lncRNA-miRNA crosstalk rather than focusing on lncRNAs in isolation. Third, it offers strategic implications for therapeutic interventions; given the systemic nature of ncRNAs, future therapies should target both lncRNAs and miRNAs simultaneously, where co-targeting miR-122 and miR-217 (cardio-metabolic) with lncRNA-miRNA hubs like NBAT1/miR-21 could offer a synergistic approach to treat tumors, overcome drug resistance, and mitigate cardiac complications associated with both disease and therapy. Ultimately, incorporating these pathways reinforces the concept of ncRNAs as systemic regulators connecting metabolism, oncology, and cardiology, paving the way for developing robust multimodal diagnostic panels and therapeutic strategies for patients with complex comorbidities.

## Conclusion

This review underscores the indispensable role of lncRNAs as molecular architects linking T2D and Colorectal Cancer. By regulating shared signaling pathways and epigenetic landscapes, lncRNAs like MALAT1, H19, and ANRIL drive the co-evolution of metabolic dysfunction and tumorigenesis. However, to fully capitalize on the clinical potential of non-coding RNAs, future perspectives must broaden the horizon to include systemic ncRNA networks. The integration of recent evidence regarding lncRNA-miRNA axes (e.g., NBAT1/miR-21, PVT-1/miR-145) and the pivotal role of miR-217 and miR-122 in cardiovascular pathology (e.g., the miR-122/Sirt-6/ACE2 axis) necessitates a paradigm shift from single-molecule diagnostics to multi-ncRNA panels. These panels offer a more robust strategy for risk stratification, enabling the simultaneous assessment of cancer progression, metabolic status, and cardiac vulnerability in T2D patients. Ultimately, therapeutic strategies targeting these interconnected networks—simultaneously modulating lncRNAs and miRNAs—hold the promise of precision medicine approaches that address the complex, life-threatening comorbidities of the modern era.
